# How do prenatal people describe their experiences with anxiety? a qualitative analysis of blog content

**DOI:** 10.1186/s12884-022-04697-w

**Published:** 2022-05-10

**Authors:** Shayna K. Pierce, Kristin A. Reynolds, Madison P. Hardman, Patricia Furer

**Affiliations:** 1 Department of Psychology, University of Manitoba, 190 Dysart Road, Winnipeg, MB R3T 2N2 Canada; 2 Department of Clinical Health Psychology, University of Manitoba, Winnipeg, Canada

**Keywords:** Anxiety, Pregnancy, Prenatal mental health, Blog, Grounded theory

## Abstract

**Background:**

Despite elevated prevalence rates of prenatal (antenatal) anxiety across studies (13–21%), and prenatal people’s use of the Internet to search for pregnancy-related information and support, research investigating prenatal people’s experiences with online mental health communication, such as blogs, is lacking. This study examined blog entries focused on anxiety in pregnancy to better understand prenatal people’s Internet discourse concerning their experiences with anxiety.

**Methods:**

A Google search using the keywords “anxiety,” “pregnant,” and “blog” resulted in *N* = 18 blogs that met inclusion criteria (public blog written in English describing a personal experience with prenatal anxiety in 250 words or more). Blog content was analyzed using a thematic analytic approach based on grounded theory principles.

**Results:**

Three main themes capturing prenatal people’s experiences with anxiety as written in public blog content were developed from qualitative analyses: 1) *etiology* (subthemes: before pregnancy, during the current pregnancy, related to a previous pregnancy), 2) *triggers* (subthemes: uncertainty, perceived lack of control, and guilt and shame for not having a normal pregnancy), and 3) *symptoms* (subthemes: intertwined emotional, cognitive and physical symptoms, in addition to behavioural symptoms).

**Conclusions:**

Our findings demonstrate a need for perinatal professionals to address anxiety symptoms and triggers in pregnancy. One way to address this may be by providing credible information regarding prenatal mental and physical health to pregnant people through online mediums, such as blogs. Bloggers often discussed experiencing a combination of emotional, cognitive, physical, and behavioural symptoms, which suggests that medical and mental health professionals should work collaboratively to provide care for prenatal people experiencing anxiety. Furthermore, Cognitive Behavioural Therapy (CBT) addresses these types of symptoms, which suggests that interventions developed or adapted to meet this populations’ needs could employ this therapeutic approach. Future research should explore the reasons why prenatal people experiencing anxiety engage with blogs, the characteristics of bloggers and readers, the impact of the blogging experience on both the blogger and their audience, and the information quality of blog content.

**Supplementary Information:**

The online version contains supplementary material available at 10.1186/s12884-022-04697-w.

## Background

For most prenatal people (those who identify as pregnant), pregnancy and childbirth pose significant life changes and challenges that are often associated with mood fluctuations. When short-lived, anxiety and worry are a natural response to the many changes and feelings of uncertainty experienced throughout pregnancy. However, prolonged anxiety that is difficult to manage can be disruptive to everyday functioning and indicate an anxiety disorder. Qualitative research exploring pregnant and postpartum people’s experiences with prenatal, otherwise known as antenatal, anxiety have identified common elements across these experiences, including help-seeking barriers, a discrepancy between pregnancy expectations and lived experience, and the importance of peer support [1–4]. One online resource that allows prenatal people to ask questions about their physical and mental health and receive advice is blogs. Blogs are an online medium where people can share self-dialogue regarding various topics or experiences, including those related to anxiety in pregnancy, and receive commentary from readers [5]. There has yet to be an analysis of the content of prenatal anxiety blogs, which was the focus of the current study.

Anxiety disorders in the Diagnostic and Statistical Manual-Fifth Edition (DSM-5) are distinguished based on the anxiety’s primary focus (e.g., worry about multiple life domains for Generalized Anxiety Disorder) [6]. For people experiencing prenatal anxiety, the primary focus of the anxiety is related to various aspects of the pregnancy, such as maternal health, childbirth, and fetal development [7]. Research has explored the prevalence of anxiety across the perinatal period, with some studies offering distinct rates for the prenatal and postpartum periods. Across such studies, prenatal anxiety has been found to have an estimated prevalence of 13–21% [8–12] and has been linked to adverse effects on both maternal and fetal health. During pregnancy, anxiety increases cortisol in the mother’s blood for prolonged periods, raising the risk of harmful outcomes such as spontaneous abortion and preterm labour [13–16]. Beyond pregnancy, anxiety may be linked to cognitive, behavioural, and emotional problems in the infant as they grow older, in addition to increasing the risk for maternal postpartum mental health concerns (e.g., postpartum anxiety or depression) [17–19]. Despite the prevalence and severity of perinatal anxiety (anxiety occurring in pregnancy and up to 12 months postpartum), postpartum depression is the only perinatal mental health condition classified in the DSM-5.

Until recently, there has been a lack of discourse around prenatal anxiety in society and the literature [9, 11, 20], which has perpetuated several of the help-seeking barriers faced by prenatal people experiencing anxiety. Limited knowledge among the general public and healthcare providers about symptoms of perinatal anxiety and treatment options, which constitutes a key component of mental health literacy, can hinder pregnant and postpartum people’s help-seeking endeavours [2, 21–24]. Research by Ponzini et al. [21] suggests that there is a lack of clarity around symptoms of postpartum anxiety disorders, with perinatal people exhibiting greater familiarity with symptoms associated with postpartum depression. Further to this, qualitative research suggests that pregnant and postpartum people experiencing anxiety may not always associate their symptoms with a perinatal anxiety disorder, which can make it difficult for them to locate information and supports that address their mental health concerns [2]. Additionally, the stigma surrounding perinatal anxiety may make prenatal people reluctant to disclose their symptoms [2, 21, 22], thereby limiting opportunities for both informal and formal supports.

The Internet is an accessible resource that can help prenatal people anonymously gather information about their symptoms of anxiety. The Internet has become an increasingly popular source of health information, with over two-thirds of Canadians searching for online health information in 2020 [25]. The Internet can help users, including perinatal people, to make informed treatment decisions by both supplementing and validating information received from healthcare providers [26, 27]. Qualitative focus group research revealed that people typically have the most health-related questions during early pregnancy and felt there were too few patient-centred prenatal visits to receive sufficient information, leading them to Google for answers [28]. This need for information is reflected across the literature, with studies showing between 86–99% of people sampled having searched the Internet for pregnancy and childbirth-related information while pregnant [29, 30].

There are a range of online resources available to prenatal people experiencing anxiety who are seeking information and peer support. Focus group research suggests that some resources, such as large-scale forums, are less preferred by perinatal people with anxiety, given that the breadth of contrary opinions can muddle evidence-based information [2]. Rather, moderated online peer-support resources that allow perinatal people to hear about the experiences of other pregnant and postpartum people and normalize their own experiences appear to be more acceptable [2]. Considering the widespread demand for pregnancy-related information, blogs may be a useful resource for pregnant people as they allow users to write about their personal experiences, ask questions, and receive answers from respondents. Blogs may provide pregnant and postpartum people with genuine depictions of pregnancy, which qualitative research suggests may help to normalize perinatal people’s experiences with anxiety [2, 4]. Blogs are unique in that while other forms of qualitative data, such as focus groups and interviews, allow researchers to explore questions that they deem important regarding prenatal people’s experiences with anxiety, analyzing blogs allows for researchers to determine which topics prenatal people want to discuss or receive information about organically, outside of the research context. With regards to prenatal mental health-focused blogs, the literature is currently limited to an evaluation of blogs focused on pregnancy loss and termination [31]. As such, research on pregnant people’s experiences with prenatal anxiety as expressed through blog posts is absent from the existing qualitative literature. Given the elevated rates of prenatal anxiety and the increased reliance on the Internet and blogs for information and support, the aim of this research was to explore how pregnant people describe their experiences with prenatal anxiety by analyzing the content in public blog entries.

## Methods

### Blog selection

The first author (SP) used Google.com (United States) to search various combinations of the keywords pregnancy (prenatal, pregnant, pregnancy), anxiety (anxious, anxiety, stress) and blog (personal blog, blog). “Antenatal” was later added as another word for prenatal, as the bloggers commonly used this term. Previous research reveals that readers typically do not look at webpages beyond the third page of Google results [32]. Thus, we only analyzed blogs found on the first three pages of each search. Twenty-six open-access blog entries accessed in March 2017 were analyzed (see Table 1 for *n* per search). Blogs were written between 2011–2017. Through consultation with and approval to proceed from the University of Manitoba Research Ethics Board, formal submission of ethics and consent were not needed as blogs are publicly accessible. This study was performed with relevant guidelines and regulations. Quotes included within this manuscript have been labelled with a participant code.

**Table 1:** Sample Size Produced from Each Key-word Search

Search Terms	*Total n*	*Eligible n*	*Not Eligible n*
Personal blogs on being pregnant and anxious	11	8	3
Pregnant and anxious blogs	12	7	5
Antenatal anxiety blogs	3	3	0
Total	26	18	8

The authors analyzed each blog entry to determine whether they met the following inclusion criteria: 1) Online public blog entries; 2) Written in English; 3) Describe personal experiences with prenatal anxiety; 4) 250 words at minimum to ensure sufficient data for analysis. Given the high comorbidity of perinatal depression and anxiety [33], blog entries describing depression and anxiety were included. Of the 26 resulting blog entries, 18 met inclusion criteria. Eight were excluded: five for having less than 250 words and three for focusing on postpartum experiences. Dialogue from the 18 useable blog entries was copied into a Microsoft Word document for analysis.

### Participants

The included blog entries were written by 18 prenatal people who were experiencing prenatal anxiety. There was variation across blogs regarding which pregnancy was discussed (e.g., first, second, or third). Some of the prenatal people described their experiences with miscarriage(s) or labour complications in a previous pregnancy. Prenatal people varied in their pre-pregnancy experiences with anxiety. Participant characteristics that were mentioned by bloggers are reported in Table 2.

**Table 2:** Characteristics of Bloggers

Blog #	Date of Blog Publication	Location	Pregnancy Discussed	Anxiety Prior to Pregnancy?
1	Not specified	Not specified	Third	No
2	April 29^th^, 2014	Canada	Second	No
3	March 9^th^, 2011	United States	First	Yes
4	November 26^th^, 2015	Not specified	First	Yes
5	April 14^th^, 2015	United States	First	Not specified
6	October 2^nd^, 2016	United States	First	Yes
7	December 6^th^, 2014	Not specified	Third	No
8	June 23^rd^, 2013	United States	First	Yes
9	April 28^th^, 2012	Not specified	First	Yes
10	April 29^th^, 2015	Not specified	First	Not specified
11	June 2^nd^, 2015	Netherlands	First	No
12	January 21^st^, 2017	United States	First	Yes
13	July 12^th^, 2016	Not Specified	Second	No
14	October 15^th^, 2014	United States	Second	No
15	November 7^th^, 2013	United States	First	No
16	August 25^th^, 2015	New Zealand	Second	No
17	May 26^th^, 2016	Australia	Second	No
18	August 17^th^, 2012	Not specified	First	Yes

### Data analysis

We completed a thematic analysis based on grounded theory principles, including the specific tenets of the coding process (e.g., specify levels of coding, memoing, etc.) [34, 35]. The identification of themes was achieved in three stages: initial coding, focused coding, and theoretical coding [36]. Initial coding involved the first (SP) and second authors (KR) independently coding each line with gerunds, in vivo codes, or direct quotes to allow us to stay as close as possible to the data and the processes being described by bloggers. Memoing was achieved during this stage by having each coder keep a coding journal to track coding decisions and preliminary thoughts regarding themes. Our coding became more conceptual as we progressed through the analysis of blog content, within the focused and theoretical coding stages. In the focused coding stage, conceptual codes were developed by comparing preliminary themes and grouping together themes from multiple blogs. During the theoretical coding stage, codes were refined and employed into a relational explanation of the main themes and subthemes emergent from the data. These stages were moved through reflexively rather than sequentially as new data was coded. Each coder keep a coding journal throughout, which acted as an audit trail for coding decisions. Coding challenges were reviewed and resolved collaboratively in line with previously established standards for rigorous qualitative research [35].

## Results

**Fig. 1:**
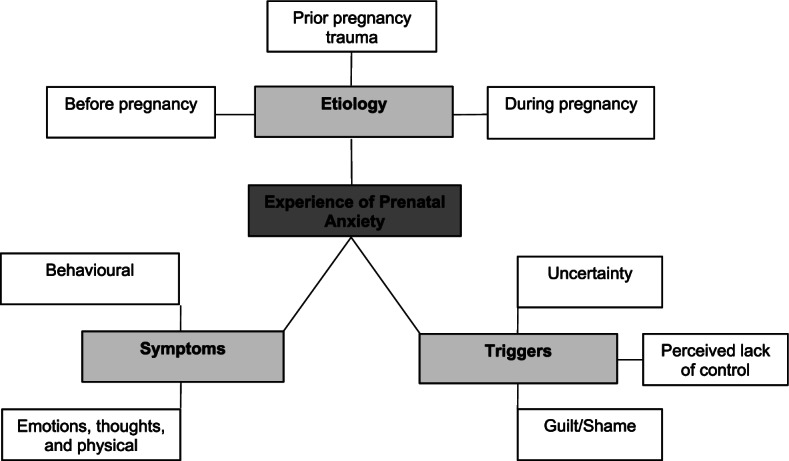
Model of themes capturing bloggers’ experience of prenatal anxiety. Dark grey represents the topic. Light grey represents main themes. White represents subthemes

Three main themes and related subthemes regarding prenatal people’s experiences of anxiety emerged from the analysis of blog entries: 1) *etiology* (subthemes of before pregnancy, during the current pregnancy, related to a previous pregnancy), 2) *triggers* (subthemes of uncertainty, perceived lack of control, and guilt and shame for not having a normal pregnancy), and 3) *symptoms* (subthemes of a combination of emotional, cognitive, and physical symptoms, in addition to behavioural symptoms). Figure 1 provides a diagram illustrating the main and subthemes. Supporting quotes for each subtheme can be found in Table 3.

**Table 3:** Supporting quotes for each main theme and subtheme

**Subtheme**	**Example**	**In-text Identifier**
**Main Theme: Etiology**		
**Prior to Pregnancy**	“I hoped I'd outgrow this someday—it was exhausting—but that level of anxiety has always been present in my life. By the time I was 28, and pregnant with my daughter, it was clearer than ever that the worry monster was still lurking.” (Blog #4)	1
**During Current Pregnancy**	“Within weeks of becoming pregnant, I started having thoughts and feelings I had never experienced before. A wave of anxiety took hold of me, tightening its grip over my mind.” (Blog #17)	2
	“Instantly, almost unbelievably, as if my mind has been simultaneously purged of its negative thoughts as I pushed my baby out, my mind returned to normal.” (Blog #17)	3
**Previous Traumatic Pregnancy**	“My fretfulness during this pregnancy has been 10 times worse as anxiety is now my constant companion. I expected to feel more anxious with baby (number two) after losing baby No. 1, but I never thought worry would be as persistent as it is.” (Blog #15)	4
	“I fear that I may die next time, that I may bleed out, tear horribly as I did with the first two, or just have something dangerous or scary happen. It takes up a good deal of space in my daily life.” (Blog #7)	5
**Main Theme: Triggers for Prenatal Anxiety**		
**Uncertainty**		
First prenatal appointment	“First Trimester: you feel awful, you have no reassurance that this will all be worth it, there’s the fear of if the baby does make it to delivery.” (Blog #2)	6
	“The thought of having to wait 4 weeks was literally crippling, and I found myself calling the doctor's office in tears several times—literally begging them to see me.” (Blog #3)	7
Baby's health	“Suddenly I felt like I was drowning in a sea of everything that could (and in my mind, WOULD) go wrong.” (Blog #3)	8
Mother's health	“I would see photos of women nearing the end of their pregnancy, see how giant and stretched out their stomachs were, and start to panic. I was already having such a horrible time breathing I couldn't imagine getting so much bigger and being able to function.” (Blog #10)	9
Miscarriage	“Thoughts turned to being obsessed with counting the movements of my baby because I thought he was dying inside me. I thought sleeping might hurt him so I used to try and stay awake all night. I was convinced he didn't want me to be his mother.” (Blog #16)	10
	“Living with anxiety during a pregnancy after a loss is like walking on a tight rope for nine months, with no safety net below, just waiting in fear that I will slip and fall.” (Blog #15)	11
Labour	“Then I'd start to imagine labour and how painful it will be, and what life with a constantly crying newborn would be like, and suddenly I felt like there was a 100-pound weight on my chest.” (Blog #10)	12
	“I have broken down now in two midwife appointments and cry whenever I think about having to do that again. I have been diagnosed with PTSD and clearly I have anxiety and depression which I have never experienced in my lifetime. I fear that I may die next time, that I may bleed out, tear horribly as I did with the first two, or just have something dangerous or scary happen. It takes up a good deal of space in my daily life.” (Blog #7)	13
Life impact	“I worried about bigger stuff, finances, how would having a baby change my marriage, will I learn the ropes of motherhood quickly enough.” (Blog #4)	14
Guilt/shame	“From the positive test to the moment that you hold your new baby in your arms, pregnancy is expected to be a time of excitement and joy.” (Blog #13)	15
	“I found it particularly difficult being with other pregnant women. It was hard to get real and relate with what they were feeling. Everyone would talk about feeling so great. I was thinking “Why don’t I feel fine?” I felt isolated when everyone else was having such great pregnancy experience and I was suffering.” (Blog #12)	16
	“I felt like I’d already failed as a mother when I was put under maternal mental health. I thought I was a terrible mother who shouldn’t be allowed to have children. I thought horrible things about myself and considered that maybe I should just leave my husband to have both children – as I was so useless they wouldn’t even notice if I wasn’t there. I thought they’d be better off if I wasn’t there.” (Blog #16)	17
**Perceived Lack of Control**		
Privacy	“I'm more terrified than ever to be around people. Why? Because nobody has any boundaries when it comes to pregnant women. The constant questions about my child's gender, name, nursery theme, baby shower, which hospital I will deliver in, whether I will have an epidural or go natural, who I will have in the delivery room—they attack a young mom like me, like a medieval army would attack an unarmed city, Swiftly and without mercy.” (Blog #6)	18
Labour and delivery choices	“I hoped and prayed that the baby would be breech so I had an excuse to not go through an un-medicated vaginal birth again. My husband assured me that I would feel disappointed in myself and the experience if I "wussed out”.” (Blog #7)	19
Bodies	“Every moment I didn’t feel nauseous was a reminder of how little I understood what was happening inside of me — and how little I could control it.” (Blog #14)	20
	“I begged my husband to let me have an abortion. He looked at me with horror and told me it wasn't an option. I had never felt so enraged. Why did I have to go through this, alone? It was my body, MY suffering.” (Blog #18)	21
Anxiety	“I try to do what I can when I can in different aspects of my life to maintain order, but there is a lurking emotional monster somewhere near me at all times. By the time I was pregnant with my daughter, it was clearer than ever that the worry monster was still lurking.” (Blog # 7)	22
	“I was buried deeper in despair than I thought humanly possible and realized the only way for me to escape would be to either terminate my pregnancy or terminate my life. This thought gave me the only feeling of hope or peace I'd had in weeks.” (Blog #18)	23
**Main Theme: Symptoms of Prenatal Anxiety**		
**Thoughts, Feelings, and Physical Symptoms**	“My brain. It’s not fully functioning right now. I walk around feeling like I’ve been drugged. I can’t concentrate, everything is muddled, and I just want to sleep.” (Blog #2)	24
	“Not too long after that, I started noticing that I was far more impatient and irritable than usual and that I just didn’t feel like my usual self.” (Blog #1)	25
	“I felt so ill-equipped to cope with normal biological pregnancy symptoms like nausea and fatigue. My serotonin had been so depleted that I didn’t have a supply to support me during the huge hormonal shifts associated with pregnancy. I had heart palpitations, difficulty sleeping, racing obsessive thoughts. It was so difficult coping with these physical symptoms of pregnancy, I was crying all the time and completely overwhelmed. I thought this was the worst thing that ever happened to me.” (Blog #12)	26
**Behavioral Symptoms**	“I didn’t feel like doing things that I usually really enjoy. Writing and blogging, for example, became suddenly very difficult and more of a chore than a hobby. I found myself withdrawing from my friends and even my husband. I realized that I preferred to be alone most of the time which is pretty atypical for me.” (Blog #1)	27
	“I temporarily lost all interest in my outside goals and pursuits as all my energy is sapped up by the work of creating another life.” (Blog #2)	28
	“Pregnancy tends to turn my whole being inwards. I become more private.” (Blog #2)	29

### Main theme: etiology

All bloggers discussed the ways their anxiety developed, which we categorized into three main etiological pathways: anxiety developing before pregnancy, during the current pregnancy, or related to a previous pregnancy. Many bloggers described having pre-existing non-perinatal anxiety and how their anxiety worsened throughout pregnancy (Table 3, quote 1). A few bloggers described developing anxiety during pregnancy, particularly during the first trimester (Table 3, quote 2). These bloggers mentioned that their anxiety levels only decreased after delivering their baby (Table 3, quote 3). Lastly, some bloggers described developing anxiety in their current pregnancy due to challenges encountered in a previous pregnancy, such as miscarriage(s) (Table 3, quote 4) or life-threatening complications during labour (Table 3, quote 5).

### Main theme: triggers for prenatal anxiety

All bloggers discussed at least one trigger for their prenatal anxiety, with many describing multiple triggers. Triggers resulting in worsened anxiety included three subthemes: uncertainty, perceived lack of control, and guilt and shame for not having a “normal” pregnancy free of anxiety.

### Uncertainty

The bloggers described six areas of uncertainty: waiting for their first prenatal appointment, their baby’s health, their health, the possibility of miscarriage, the experience of labour, and how having a baby would impact their life. Many bloggers described feeling the most uncertain during their first trimester (Table 3, quote 6). Uncertainty sprouted from having to wait 8–10 weeks into their pregnancy for their first prenatal appointment (Table 3, quote 7) and uncertainty around their baby’s health. Health concerns included their babies’ development, rare medical conditions that their baby might have, and symptoms that might indicate pregnancy complications (e.g., abdominal cramps creating concern of ectopic pregnancy). The mere thought of their baby not being healthy triggered anxiety (Table 3, quote 8). Additionally, many bloggers discussed how uncertainty around how the changes occurring to their body would impact their physical health triggered their anxiety (e.g., difficulty breathing as their stomach grows throughout pregnancy; Table 3, quote 9).

A few bloggers discussed how the fear of miscarriage triggered their anxiety. For some, this fear pertained to whether their regular activities, such as sleep, would harm their developing baby or even cause a miscarriage (Table 3, quote 10). For others, this fear involved previously experiencing a miscarriage (Table 3, quote 11). Thoughts regarding the experience of labour also triggered anxiety (Table 3, quote 12). For one blogger, this was due to their experience in their previous labour (Table 3, quote 13). Lastly, many bloggers expressed uncertainty regarding how having a baby would affect aspects of their lives, including their relationships, finances, and whether they could learn how to be a parent (Table 3, quote 14).

### Perceived lack of control

Related to the aforementioned areas of uncertainty, many bloggers expressed a perceived lack of control over their privacy, their choices regarding labour and delivery procedures, the various changes happening to their bodies, and their anxiety during their pregnancy. Regardless of the cause, bloggers expressed that their perceived lack of control increased their prenatal anxiety. Concerning privacy, one blogger described feeling attacked in social interactions when asked intrusive questions about their pregnancy (Table 3, quote 18). Some bloggers expressed feeling a lack of control over their bodies, particularly over choices related to labour and delivery procedures (e.g., whether to have an unmedicated or medicated labour and delivery; Table 3, quote 19), the changes occurring to their bodies to support the developing fetus (Table 3, quote 20), and deciding whether to continue their pregnancy (Table 3, quote 21). Lastly, a few bloggers described feeling they lacked control over their anxiety during pregnancy, referring to it as negative and uncontrollable (Table 3, quote 22). For a few bloggers, their anxiety elicited such distress that they felt that termination of the pregnancy or suicide was their only option (Table 3, quote 23).

### Guilt/shame for not having a “normal” pregnancy

A few bloggers elaborated on the stigma surrounding what emotions prenatal people ‘should’ feel during pregnancy (Table 3, quote 15). Bloggers described how experiencing negative emotions during pregnancy, rather than the expected positive emotions, triggered their anxiety. Many felt guilt and shame for not having a “normal” pregnancy filled with positive emotions (Table 3, 16). For a few bloggers, this guilt and shame led them to question whether they would be good mothers (Table 3, quote 17).

### Main theme: symptoms of prenatal anxiety

Discussion of the symptoms of anxiety that bloggers experienced during pregnancy revealed two categories of symptoms commonly experienced during the first trimester: 1) An inter-woven experience of emotions, thoughts, and physical symptoms; and 2) Behavioural symptoms. Emotions and thoughts described by bloggers included feeling selfish, negative, guilty, ashamed, embarrassed, disconnected, fretful, worried, impatient, and intolerant (Table 3, quotes 24 and 25). Bloggers discussed these in conjunction with physical symptoms, including changes to their bodies that increased anxiety (i.e., hormone changes, body changes) and physiological aspects of anxiety (i.e., irregular breathing, heart palpitations, panic, and difficulty sleeping; Table 3, quote 26). Lastly, many bloggers discussed their behavioural symptoms, including withdrawing from conversations and relationships, isolating, and pulling back from previously enjoyed activities and goals (Table 3, quote 27, 28, and 29).

## Discussion

The findings from this study are additive and complementary to the existing qualitative literature focused on prenatal people’s experiences with anxiety. The themes captured in this study reflect common themes highlighted across the literature, including stigma around perinatal mental health concerns and pregnancy as a period of uncertainty, in particular during the first trimester [1, 2, 4]. Of note, this study is the first to examine prenatal people’s experiences with anxiety using online public blog entries. Though it was beyond the scope of the current study, in terms of objective and methodology, to explore why bloggers chose to use blogs as a means to share their experiences, based on the content shared across blogs, there appeared to be a desire to share information to make meaning from experiences, to offer support to others such that they may feel less alone in their experiences, and to obtain support in comments and feedback on the blog itself. Following a thematic analytic approach based on grounded theory principles, we identified three main themes evident to the online discourse presented in 18 prenatal anxiety blogs analyzed: *etiology*, *triggers*, and *symptoms of prenatal anxiety*. 

Regarding etiology, most bloggers reported anxiety before becoming pregnant or noted having developed anxiety due to previous pregnancy experiences. This highlights the importance of and need for early detection and intervention to prevent prolonged prenatal anxiety and the associated adverse consequences for the mother and developing fetus [13–16]. Further, early detection and intervention are suggested to be helpful for prenatal anxiety given the lack of understanding among the general public of what constitutes normal versus abnormal anxiety in pregnancy [12]. With regards to triggers, the most common trigger to anxiety in the first trimester was a perceived lack of control, which was distinct from but often discussed in conjunction with uncertainty regarding the blogger’s health and that of their developing fetus.

Uncertainty concerning upcoming labour and delivery was also commonly discussed, especially by those who experienced prior labour and delivery complications or traumatic events. Bloggers often discussed uncertainty in the context of the first trimester, with many noting that the 8 – 10 week wait-time for their first prenatal appointment contributed to feelings of anxiety. Intolerance of uncertainty is a key component in the development and maintenance of anxiety disorders, thereby highlighting the need for early access to prenatal medical and psychological care in order to create opportunities for prenatal people to voice their concerns and receive prenatal education and support [37, 38]. Our findings pertaining to uncertainty are consistent with those of previous studies, highlighting the ways in which maternal health and birth outcomes serve as triggers for anxiety and pregnancy-specific stress [4, 39–41]. While blogs allow for prenatal people to obtain experiential based answers to their questions concerning the wide range of uncertainties experienced in pregnancy, there is room for healthcare providers to engage with blogs by commenting on existing blogs. Such engagement may help bloggers navigate uncertainties by providing validation and credible information and resources around prenatal physical and mental health. Further, given the increasing use of the Internet by prenatal people to find health information [29], developing a comprehensive and credible online resource, such as a blog, that focuses on the maternal prenatal experience could address these uncertainties. Within focus group research, perinatal people have voiced a need for online evidence-based information as well as moderated peer-support resources, suggesting that a clinican-led blog containing both of these elements would be well-received by this population [2]. An online resource could help to provide information to pregnant people before their first prenatal appointment, thereby having implications in anxiety management. Interventions that aim to improve intolerance of uncertainty may also help to mitigate anxiety throughout pregnancy.

Various prenatal anxiety symptoms were mentioned by bloggers, most of which were described as disrupting their daily functioning. These symptoms overlapped with several characteristics of clinical anxiety, as outlined in the DSM-5 [6]. Many of these symptoms, such as fatigue, panic attacks, lack of emotional control and excessive worry, have all been shown to evoke negative consequences for both maternal and fetal health [13–16]. These results support the need for increased assessment and treatment of prenatal anxiety, particularly early in the prenatal journey given that symptoms were most distressing in the first trimester. This aligns with research findings that prenatal anxiety is highest for primiparous mothers during early pregnancy, further highlighting the need for early detection of prenatal anxiety [42]. Challenges associated with early detection and treatment of prenatal anxiety symptoms include perinatal people’s preference to seek informal supports (e.g., friends or family), disconnected communication between healthcare providers, and limited availability of mental health resources [22, 43].

The complex emotional experiences described by the bloggers further demonstrate this need. Bloggers’ discussion around feeling guilt and shame for not having a “normal” pregnancy reflects the lack of recognition of prenatal anxiety, as reported in previous literature [44]. This finding aligns with the existing qualitative literature, which has found that mothers experience higher levels of anxiety during their first pregnancy as they navigate greater levels of uncertainty around what can be expected, both physically and emotionally, throughout pregnancy [4]. The lack of societal discussion and clinical recognition of perinatal anxiety does little to reduce the stigma surrounding anxiety during pregnancy. Stigma may explain why many bloggers experienced guilt or shame over their symptoms and chose not to share their negative feelings with their friends or family. Qualitative findings suggest expecting mothers are often hesitant to disclose their symptoms to loved ones or healthcare providers given societal expectations that pregnancy should be a joyous time [1, 3]. Recognizing prenatal anxiety as a clinical disorder may decrease the guilt and shame experienced by many prenatal people experiencing anxiety and may promote effective support seeking.

### Limitations

Researchers, practitioners, and clinicians should consider these novel findings along with their limitations. The analysis of public blogs meant that we could not determine our sample’s diversity, as blogs did not always mention sample characteristics. Contextualizing the sample is important, as prenatal people of varying socioeconomic statuses and sociodemographic backgrounds may experience prenatal anxiety differently. Furthermore, though not an objective of the current study, we were not able to follow-up with blog authors to explore their reasoning for engaging with blogs and ask clarifying questions, as bloggers’ contact information was not publicly available. Future research should explore why pregnant people engage with blogs, in addition to gathering more information about the characteristics of prenatal anxiety bloggers. There are also several limitations associated with the search strategy that we employed. We used Google to complete our search, as over 70% of Internet searches are conducted using this search engine; however, other search engines may have yielded different results [45]. Furthermore, we also only analyzed blogs that were written in English. Those written in other languages may contain different perspectives on anxiety in the prenatal period. Additionally, our search was conducted in 2017 and does not include more recent blogs that may have been posted on prenatal anxiety. An updated search may expand upon the thematic framework developed within the present study by revealing additional themes or nuances within created themes. As with all studies involving websites and blogs, online content changes rapidly; however, our objective was not to conduct an exhaustive search of all prenatal anxiety blogs, but rather, to provide a preliminary understanding of prenatal people’s experiences with anxiety as expressed through blogs, which are a relatively novel source of qualitative data within perinatal mental health research. Despite these limitations, utilizing public blogs is a novel advancement of qualitative methodology that offers access to people’s perspectives while limiting research bias.

## Conclusion

This is the first study to examine public blog entries to gain a rich understanding of prenatal people’s experiences with anxiety. The main themes uncovered through our analyses reveal important implications for perinatal professionals such as family physicians, obstetricians and gynecologists, nurses, and mental health professionals. First, these findings demonstrate a need for perinatal professionals to address misinformation and the misconception that a ‘normal’ pregnancy does not include negative emotions. Increasing the provision of credible information to perinatal populations around healthy fetal development, the breadth of physical and emotional changes commonly experienced in pregnancy, recognition of perinatal mental health symptoms, and the types of professionals who can help may serve to address these triggers. This information could be provided to prenatal people digitally in the form of a blog or through comments on existing blogs. Second, prenatal people’s engagement with online blogs highlights to perinatal professionals that the Internet is an accessible, commonly used resource for this population and suggests that online information, peer support, and treatments for perinatal mental health could be useful and are needed. Third, bloggers often discussed experiencing a combination of emotional, cognitive, and physical symptoms in conjunction with behavioural symptoms, which suggests that interdisciplinary professional teams (i.e., medical professionals and mental health professionals) may be most effective when caring for people experiencing prenatal anxiety. Furthermore, these types of symptoms are commonly addressed through Cognitive Behavioural Therapy (CBT), identifying important implications for the development or adaptation of interventions for prenatal anxiety.

Future research involving prenatal anxiety blogs should aim to better understand the characteristics of prenatal people using blogs, the reasons bloggers and readers engage with blogs (e.g., to share their experiences, to seek support from peers), the impact of the blogging experience on both the blogger and their audience, and the quality of information that is being shared. Additional areas for future research include an exploration of the information and intervention needs of prenatal people experiencing anxiety using a mixed-methods approach, such as surveys including open and closed questions to assess endorsed anxiety symptoms, severity of symptoms, and the impact of prenatal anxiety among a broader sample. By fully understanding the experiences and needs of pregnant people, researchers, practitioners, and clinicians can develop more effective assessment and intervention techniques, or tailor already-established anxiety interventions, such as CBT, to this population’s needs.

## Supplementary Information

**Additional file 1.**

## Data Availability

All data generated or analysed during this study are included in this published article [and its supplementary information files].
