# Supplementary material for: How do prenatal people describe their experiences with anxiety? a qualitative analysis of blog content
Source: BMC Pregnancy Childbirth. 2022 May 10;22:398. doi: 10.1186/s12884-022-04697-w (PMC9092700; doi:10.1186/s12884-022-04697-w)
Supplement: Supplementary file 1 — Additional file 1. [file 12884_2022_4697_MOESM1_ESM.docx]

How do Prenatal People Describe their Experiences with Anxiety? A Qualitative Analysis of Blog Content

**Content from Blog #1**

*This post is not intended to diagnose or treat any symptoms. If you feel this is something you are dealing with, it’s very important that you talk to your doctor or midwife about it as soon as possible.*

Typically when women find out they are pregnant, there is excitement and anticipation for their upcoming bundle of joy.

But what happens when those feelings of excitement are masked by feelings of overwhelmedness, anxiety and depression?

**My Experience**

I found myself in that situation recently when my husband and I learned we were expecting our third child.  I suspected I was pregnant fairly early – at about 4 weeks.  Not too long after that, I started noticing that I was far more impatient and irritable than usual and that I just didn’t feel like my usual self.  These feelings seemed above and beyond the typical changes that happen during pregnancy.

I didn’t feel like doing things that I usually really enjoy. Writing and blogging, for example, became suddenly very difficult and more of a chore than a hobby.

I found myself withdrawing from my friends and even my husband.  I realized that I preferred to be alone most of the time which is pretty atypical for me.  And I felt so anxious!  Anxious about the baby’s development, anxious about the delivery, anxious about the months following the delivery.  You name it and I probably worried and fretted about it

The worst came when I realized, upon waking in the mornings, that I was discouraged before even getting out of bed.  I dreaded that I had to face another day of morning sickness and sad, anxious feelings.  Day after day I wanted to pull the covers up over my head and sleep – sleep until this whole mess was over and I could be “normal” once again.  That’s when I knew that I needed help.

**Having these anxious and depressed feelings doesn’t mean that I don’t want my baby.** In fact, nothing could be further from the truth.  I am excited about holding my sweet little one, seeing what he or she will look like and getting to know that new little personality.

But, discouragingly, I find it difficult to enjoy those joyful feelings with all the other negative thoughts swirling around in my head.  And all of that in addition to the typical discomforts of early pregnancy. **I find myself being discouraged that I can’t just be happy, and that creates a cycle of guilt and negative feelings which is difficult to defeat.**

I also realized that I had to overcome the stigma of depression, especially in my own mind.  Even though it is more widely known and understood than ever before, I still struggle with these feelings because I feel like it is a weakness that I should be able to easily “fix”.

**Even now, I feel embarrassed and ashamed to admit, even to myself, that I’m having these feelings and emotions.** I don’t want to be depressed, but the more I tried to ignore it, the more depressed I became.

**Depression during pregnancy?**

Most people are familiar with post-partum depression, which is a type of depression which affects new mothers in the year following the birth of their baby.  Doctors and midwives take great care in asking their patients, who have recently delivered, whether they are noticing any symptoms of post-partum depression brought on by the sudden change in hormones after delivering.

**Depression *during* pregnancy, called antenatal depression, is much less well known.** In fact, I’m not sure I even knew there was such a thing until it happened to me.

Now as I look back, I can see signs of some depression and anxiety during each of my pregnancies.  I also struggled with post-partum depression but didn’t realize that until after the birth of my second child.  My symptoms and the effect of the depression seem to be getting worse with each pregnancy, perhaps because it went unnoticed and untreated for so long.

**Symptoms of antenatal depression:**

In addition to the normal effects of pregnancy such as fatigue and needing to sleep more, you may be suffering from depression if you have some of these symptoms as well:

- Persistent feelings of sadness
- Lack of interest in hobbies and other favorite activities
- Difficulty concentrating and making decisions
- Persistent feelings of anxiety and anxious thoughts
- More irritable and impatient than usual
- Anxiety about the pregnancy and delivery
- A sense that nothing seems enjoyable or fun anymore, including the pregnancy
- Thoughts of death or suicide

**What causes antenatal depression?**

Similar to post-partum depression, antenatal depression is thought to be triggered by sudden and intense hormonal changes.

Other factors that might contribute to antenatal depression are: a personal or family history of depression, relationship difficulties, stressful life events and problems or complications with your pregnancy. However, there is still much that is unknown and needs to be researched in this area.

**Treatment for antenatal depression**

**Whatever the cause, the important part to focus on is the treatment of antenatal depression.** Even while pregnant there is much that can be done, with the help of qualified doctors and midwives, to ease the symptoms and have you feeling back to your normal self again… well, your normal pregnant self.  :)

**Simply knowing what you are dealing with is often half the battle.**

Once I finally acknowledged that something was wrong and I talked to my doctor, I felt better.  **I knew what I was up against, I had a treatment plan and so I felt better, if only because I felt that I was being proactive.** Wallowing in depression will only drag you further down.  But facing and fighting it head on will help you to keep moving, even on the most difficult days.

**Establish a strong support system.**

With the help of my husband, my mom, my sisters and some close friends, I have a strong support system of people I can call upon when I’m feeling low.  Talking through my thoughts and feelings always helps and often it’s just nice to talk about something other than the difficulties and depression.  **Surround yourself with people who love you, support you and those you can make you laugh.** Laughter is very good medicine!

**Try therapy or counseling.**

Since I recognized these feelings somewhat from my earlier pregnancies and post-partum experiences, I knew they were severe enough this time that I needed some help.  I found an excellent counselor who practices not far from my house.  The first time I went to visit with her, I was nervous.  What if I didn’t like her?  What if she didn’t like me?  What if she told me I was making all this up?

Of course I had nothing to worry about and I left her office that first day feeling 10 pounds lighter.  **I’ve found it nice to have a professional opinion about all those negative thoughts swirling in my mind and she has some excellent suggestions for me on how to deal with those thoughts.** I feel that I’m building up a tool belt of ideas that I can use to address the anxious and depressed feelings I find myself having.

**If worse comes to worse, there’s medication that can help.**

Honestly, I really struggled with this decision and it weighed heavily on my mind for some time.  My doctor felt that my depression was severe enough to benefit from the help of some anti-depressants.  But during my previous pregnancies, I rarely even took so much as Tylenol so I felt really guilty for considering anti-depressant medication.

After much prayer and discussion with my husband, we decided that the benefits outweighed the risks.  For one thing, stress hormones are not good for you or for your baby and I was certainly generating more than my share of those.  In addition, I did not feel I was being a good mother to my other children in my depressed and anxious state.

For these reasons we decided it was best for me to take the anti-depressant medication and my doctor recommended one that has been proven safe to use during pregnancy.  **It’s a very personal decision that must be made with the help of qualified doctors or midwives and also with much thought and prayer.** What’s right for one isn’t right for all but it’s nice to know that there are options should the depression and anxiety be severe enough to warrant it.

**Some Additional Thoughts**

- **Don’t be ashamed if you are suffering from depression**.  Unfortunately, depression itself can often cause you to feel that you are weak or that you should be able to control your feelings better.  This may result in comparing yourself to those who do not suffer depression and assume that they are doing it right while you are doing it wrong.  These things are false.  Depression is real and it is not your fault.  The first step to feeling better is not being ashamed to do something about it.
- **Don’t be afraid of therapy.** Unfortunately there is also a stigma in our culture surrounding therapists.  But I can tell you from experience that they can bring a huge sense of relief and help both to give you the tools to help you deal with depression as well as help you to realize that your problems are normal so that you can stop blaming yourself.  Do a little homework to find a therapist that will be able to help you in a way with which you’re comfortable and then get help!
- **Don’t blame yourself.** As I mentioned earlier, it is common to have feelings like “this is all my fault”, or “Other people can handle this situation, what’s wrong with me?”.  Toss these feelings out.  One of the things I’ve learned from my therapist is that, just because I feel a certain way doesn’t mean that it’s reality.  If you give yourself a break and stop beating yourself up, you will find a hidden reservoir of time and energy and peace.

I’m now over 11 weeks pregnant and the weeks leading up to this point have definitely been a roller coaster.  I do wish that I could have a glowing, happy, anxiety-free pregnancy.  But for one reason or another, it’s just not that way for me.

I’m learning that it’s best for me to look forward and remind myself that although these times are difficult, they won’t last forever.

**Have you been depressed while you were pregnant?  What are some things that helped you to cope during that difficult period?**

**Content from Blog #2**

I’ve started so many blog posts in the last couple of weeks, but haven’t had the energy or mental clarity to finish any of them. So I thought perhaps it was a time for a personal update. Which of course involves an update on the pregnancy. (I promise these won’t take over the blog.)

**I’ve been having a hard time writing about it for a couple of reasons.**

1. My brain. It’s not fully functioning right now. I walk around feeling like I’ve been drugged — I can’t concentrate, everything is muddled, and I just want to sleep. I don’t remember this with my last pregnancy. I can’t write more than three sentences without wanting to go lie down. (This post was written in stages).

2. One thing I *do* remember from my first pregnancy is that carrying a child tends to turn my whole being inwards. My introverted tendencies get ramped up and I become more private. At least in the first trimester, I temporarily lose all interest in my outside goals and pursuits as all my energy is sapped up by the work of creating another life.

3. When you talk a lot about infertility, you end up building connections with a lot of other people going through the same thing. And then if you end up getting pregnant, it gets kind of awkward.

Because you know how heart-wrenching it is to get pregnancy news when you’re still waiting. You just *know* you’re causing someone pain. You’ve been there yourself. So it’s hard to talk openly about it. You don’t want to talk about how awesome it is, because then you’re just rubbing it in their faces; but you also don’t want to talk about how hard it is, because then you’re being ungrateful for this incredible blessing you’ve been given.

But of course it’s still important to talk about it. I want to be honest and open. (I can’t *not* be, I guess. It’s a disease.)

**So here’s where I am with my pregnancy.**

(I’m at 10 weeks).

It’s been a long month. I’m tired all the time, and until recently, have been pretty nauseous most of the time. (But not throwing up, thank goodness.) Food is my nemesis. Even on good days, I get little pleasure from food. The smell of the fridge interior repulses me. Cooking is a nightmare.

I praise the Lord daily for my wonderful mother, who has brought us more meals than I can count and saved us from starvation (or at least bankruptcy from all the Vietnamese takeout.) On days when we aren’t given food, I heat up a frozen pizza or cook store-bought perogies. Yesterday for lunch I got *dangerously close* to picking up Arby’s, but then [name de-identified] suggested we try the local cafe and we were saved by vegetarian ciabatta sandwiches.

I’m just starting to feel better, though, and am hoping I will be back to regular cooking soon. I miss real food much.

**But food hasn’t even big my biggest adversary.**

**It’s been anxiety.**

Here’s the thing with the first trimester:

1. **You feel awful:** nauseated, bloated, tired, foggy, constipated, breathless, etc. All you want to do is eat and sleep and puke. All at the same time. But

2. **You have no reassurance that this will all be worth it.** The baby’s chance of survival at this point is only like 75%. There’s a good chance you’ll go through all this and more and end up where you started: without a baby.

**And then there’s the fear of if the baby does make it to delivery.**

This anxiety is especially heightened for me because [name de-identified] and I both have genetic problems in our immediate family.

I had a brother who died after three days due to a severe genetic disorder which prevented his bones from fully forming. He suffocated to death because his rib cage couldn’t support his lungs. All my parents could do was watch and grieve.

[Name de-identified] has a brother with autism (and also two cousins, one of whom passed away in childhood.) Twenty-three years later, he still brings his mother to her knees with exhaustion on a daily basis.

What kind of baby could I be carrying?

If he/she makes it, will the burden of caring for him/her weigh me down from being a good mother to [Name de-identified] (to say nothing of the child him/herself)?

At this point I haven’t heard a heartbeat. I haven’t seen an ultrasound. I just saw a plus sign on a pregnancy test and then started feeling crappy for six weeks solid.

There haven’t been any bad signs whatsoever; but nor have there been any real reassuring or good signs, either.

* * *

I go to see my midwife for the first time tomorrow. Hopefully I’ll get to hear a heartbeat, and hopefully that will inspire some more hopeful feelings.

I feel a little crappy talking to God about it, because he finally and miraculously gave me my heart’s greatest desire, and all I can do is worry.

I feel selfish asking for a perfectly healthy baby when my own parents — and countless others — have had to suffer.

**So this is where I’m at.** I feel like I’m in a kind of limbo, just waiting. I have no idea what my life will look like in seven months. Waiting around, feeling exhausted, sickish, guilty, worried, and occasionally hopeful and even excited.

I hope to be back soon with more interesting, less depressing words. I just wanted you to know where I was.

**Content from Blog # 3**

Man, all this baby talk sure is fun!  :)  Sorry for the cliffhanger yesterday – I didn’t want to blab on for so long that I made you all fall asleep. So I will just pick up right where I left off – where was I?  Ahh yes, peeing on a stick…

The funny thing is, I had done so many pregnancy tests over the past year, that I didn’t even bother to look at the box and see what I was supposed to be looking for.  Most of the ones I had done in the past were supposed to be plus signs, so when I looked at the stick and saw two bold blue lines side by side, I didn’t think anything of it.  In fact, I thought it was negative.

As I went to toss it in the trash, I saw the box peeking out from the trash can, and pulled it out just to take a second look.  It was only then that my heart skipped a beat.  I looked at the box, I looked at the test, I looked back at the box.  And then I completely freaked out.

I stood in the bathroom for about 30 seconds, totally unsure what to do next.  I had no plan, and my mind was racing so quickly!  I ran back into the dark bedroom and stood next to the bed.  The conversation that followed when something like this…

[Name de-identified]: [Name de-identified], wake up.
[Name de-identified]: What’s wrong?
[Name de-identified]: I think I’m pregnant.
[Name de-identified]: Why do you think that?
[Name de-identified]: Because I just took a pregnancy test.
[Name de-identified]: Just now?  What did it say?
[Name de-identified]: That I am PREGNANT! Go look at it!

My initial reaction upon seeing the test myself was such shock and panic that it initially outweighed any excitement.  When people ask, “were you surprised?” – I have to think, no matter how hard you try or plan, you will *always* be surprised to find out you are pregnant.

What followed our conversation is one of those moments that I know will be etched into my memory forever.  I will never ever forget the look of happiness and excitement on [Name de-identified] face.  The minute I saw his smile, I crawled back into bed, stopped being scared, and started dreaming about the possibilities.  The two of us talked under the covers for at least an hour, until we finally went up to the office so I could immediately start researching.

The very first thing I did was Google an online due date calculator, and came up with this…

[image removed to de-identify]

OCTOBER?  I was shocked!  For some reason the calculations in my head led me to believe that I was going to have a Thanksgiving baby, but somehow I had already lost a full month!  October seemed so SOON.  I’m still not totally sure how all that works – apparently you are already 2 weeks pregnant at conception?  News to me!

Next I g-chatted a friend and asked her for a doctor recommendation (although I didn’t give a reason).  Being new to the area, I didn’t have one yet – setting off a whole other string of panic attacks and freak outs.  With a doctor name and practice recommended, I picked up the phone and dialed to make my appointment, wanting as much information as I could possibly get right away.

Nurse: Hello, how can we help you?
[Name de-identified]: I need to make a new patient appointment with Dr. D.
Nurse: Okay, she can see you on March 10th (this was February 7th).
[Name de-identified]: Oh I’ll need to get in sooner than that, I’m actually PREGNANT!  
Nurse: (not sounding impressed) Oh, okay then.  Well that actually works out perfectly, because we won’t see you until you are 8-10 weeks for your first prenatal visit.
[Name de-identified]: (panicking) EIGHT weeks? Oh wow – I can’t possibly wait that long.
Nurse: Well that is standard practice.  We’ll see you on the 10th!  Congratulations!

An entire month of nothing but WAITING.  I don’t really talk about it much on here since it doesn’t affect my day to day life ([Name de-identified] would argue this), but in the past I have suffered from very bad anxiety.  I worry and panic about *everything.* It has gotten better in recent years, as I’ve made a conscious effort to recognize and manage the symptoms, but pregnancy has definitely brought back a lot of those old feelings and worries.

The minute I started researching pregnancy, I went from excited to TERRIFIED.  Every website I logged into said things like “Congratulations on your pregnancy!  Here’s what you should know about abdominal pain, risks of ectopic pregnancy, miscarriage, placental abruption, etc.”  Suddenly I felt like I was drowning in a sea of everything that could (and in my mind WOULD) go wrong.

[
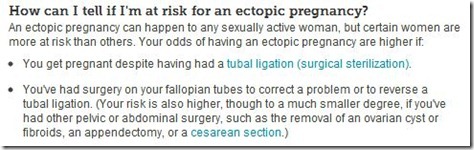
](http://www.dailygarnish.com/wp-content/uploads/2011/03/ectopic.jpg)

I spent the first week feeling somewhat crampy and uncomfortable, and absolutely CONVINCED that I was having an ectopic pregnancy.  When I was 15, I had surgery to have an ovarian cyst removed, and since this was listed as a possible risk factor for ectopics, I just knew that I had one.  The thought of waiting four weeks for answers was literally crippling, and I found myself calling the doctor’s office in tears several times – literally begging them to see me.

Finally they agreed to let me come in for some blood work to see if my hormone levels were progressing as they should.  I practically jumped in the car right then and there, off to give my blood sample for day one.  Two days later, I went back for round two – and anxiously awaited a phone call to let me know the results.  The next day, a nurse nonchalantly called with the following, “Your test results are back.  Congratulations, you are pregnant!”

Um yes, I already knew that.

After several more questions and prompting, she located the second round of numbers and told me that everything looked great, hormone numbers had more than doubled, and I was off to a healthy start.  HUGE DEEP BREATH.

Once I had SOME (any!) sort of medical confirmation that things appeared healthy and normal, I was able to stop obsessing so much about what might go wrong.  As much as I wanted to educate myself on pregnancy, I found that the resources online (and the commenters!) all tended to focus heavily on risks, possibilities, and potential negatives.  While I realized it was important for me to know the whole picture, taking into account my personal struggles with anxiety – it was way too much for me.

Around the six week mark (and after two weeks of driving [Name de-identified] insaaane with my obsession with miscarriage), I made it my personal mission to simply allow myself to be excited.  I knew that there was nothing I could do to cause or prevent any possibilities, so worrying about them was entirely pointless.

Which brings me to tomorrow – **my first official prenatal appointment**!  To say that I am nervous is an understatement, but I am hopeful and excited – hoping to hear a heartbeat.  While the idea of being pregnant already feels very real, I know that seeing and hearing this little miracle in person will take it to a whole new level.

I promise to update to you guys once I know something!  Please say a few extra prayers tonight for me, [Name de-identified], and the raspberry.

After what is hopefully a successful first appointment in the morning, we are heading to [Location de-identified] for the weekend to take care of some family business. (If I have any [Location de-identified] readers interested in getting together Saturday morning, shoot me an email!)  See you soon from the [Location de-identified]!

**Content from Blog #4**

I have always been an anxious person.

As a kid, I remember lying awake whenever my parents were out past my bedtime, waiting for the sound of the garage door to signal that they had returned home. I couldn’t drift off until I was sure they were back, safe and sound. I hoped I’d outgrow this someday — it was exhausting — but that level of anxiety has always been present in my life. By the time I was 28, and pregnant with my daughter, it was clearer than ever that the worry monster was still lurking.

From the moment that second line appeared on the stick, I could feel the tension mounting. I struggled to control my anxious thoughts and feelings, obsessing over the bean in our ultrasound photo, unable to stop myself from googling potential complications. I worried about the big stuff — finances, how having a baby would change my marriage, if I would learn the ropes of motherhood quickly enough — but mostly, I was consumed by obscure medical conditions and their symptoms. After I noticed I had strangely itchy palms for two days, I moved up my next prenatal appointment so that the doctor could confirm I didn’t, in fact, have a rare liver condition I’d read about. When I finally admitted to my obstetrician that I no longer felt the joy of pregnancy, only the dread of the “what ifs,” she referred me to a psychiatry clinic specializing in prenatal and postpartum anxiety and depression.

[Name de-identified], a perinatal psychiatrist and head of the Maternal Infant Program and Perinatal Psychiatry clinic at [Hospital name and location de-identified], says some degree of anxiety during pregnancy is normal, given the huge life changes ahead. “But,” she says, “it gets into the pathological range when it starts to become the major focus of what you’re going through, rather than just a sidebar. If the anxiety impacts your ability to function in day-to-day life, or if you feel you can’t control or put a lid on it, it’s excessive.”

Symptoms of antenatal anxiety range from compromised sleep and an inability to concentrate, to physical manifestations such as tightness in the chest, dizziness or heart palpitations. Prescribed treatment depends on the severity of symptoms. Minor levels of anxiety can be managed with talk therapy, says [Name de-identified]. “It could be as simple as talking through your concerns with someone, or it could be more formal, like cognitive behaviour therapy. Exercise and yoga are also good ways to handle lesser anxiety.” More serious anxiety may require medication, she says.

**Video on prenatal yoga is found here (see transcription below)**

One technique that worked for me was keeping an anxiety journal. List your anxious thought, how it makes you feel, and the evidence for the worry (for me, it was symptoms I was experiencing) and against (I allowed myself to look at reputable health websites only — just the facts). Seeing my concerns mapped out on paper made them seem less scary. [Name de-identified] calls this “walking yourself back to the middle.”

Women who have a history of anxiety should be extra careful to monitor their mental health during pregnancy. “While it’s possible that someone who has never experienced anxiety before will develop it during pregnancy, people who have anxious personalities, or who are perfectionists, are at higher risk,” [Name de-identified] says. She reports that women usually return to their baseline postpartum.

This was (mostly) true for me. While I still stress about the health and development of my now-19-month-old daughter, [Name de-identified], I’ve learned to cope with the “what ifs” along the way. Now I worry that if I don’t stop worrying I’ll miss out on the best parts of her life. And that’s a double negative if I’ve ever heard one.

**Video in the Blog Transcription:**

There’s many benefits for prenatal yoga. Umm one of them definitely is it helps alleviate some of the common symptoms of pregnancy, it helps relive any stress and sort of ease the mind of the mom to be. Also there is many poses that really prepare you for the labor so helping tone the pelvic floor, helping your body become strong, opening the hips, and a lot of these postures that are doing all this benefit before the birth are actually going to help during labor as well.

If you want to start prenatal yoga usually you want to speak with your doctor and make sure that you don’t have any conditions that maybe you’re not able to do certain postures. If that’s the case then we’ll know that starting out, you can inform your teacher which would be a certified prenatal yoga teacher and we can work with you from there. So there may be certain postures that you modify or other postures that you may not participate in. But you’re definitely welcome to do the prenatal yoga

**Content from Blog #5**

Today is off to a much better start than yesterday. Truth be told, yesterday was a bit of a rough one for me due to some pregnancy-related anxiety. *Everything is okay*, but I was just a hyper-concerned mama-to-be for most of the day. I know I’ve alluded to the anxiety I’ve felt during pregnancy on the blog before, but yesterday it surfaced again worse than it has before.

On Sunday, a couple hours after brunch, I started to experience some cramping. This happened to me one other time many weeks ago during pregnancy right before bed and by the time I went to sleep and woke up the next morning, everything felt okay again. I just sort of figured this would be similar to my last cramping experience, but when the cramping didn’t dissipate for hours and I woke up on Monday with a continuous light cramping feeling, I started to feel a little concerned. I went about my normal work day feeling overly aware of a light cramping sensation that never let up. The cramping also seemed to migrate lower and lower which heightened my anxiety.

I started to get myself more and more worried (why I chose to consult Dr. Google when I *know* that will just freak me out further, I’ll never know) and eventually called my mom after work to explain how I was feeling. My mom is a nurse and asked me a handful of questions and said she thought I was fine but encouraged me to call my OB/GYN just to be safe. I spoke with a triage nurse who asked me a bunch of very thorough questions. She was so kind and calm and by the end of our call, she said she thought my cramping was likely gastrointestinal versus uterine (which she said was a good thing) and asked me to go to a local CVS or Walgreens for a quick blood pressure screening, which I did immediately. She also told me she thought my cramping could be related to dehydration and encouraged me to drink lots of water.

Once I arrived at CVS, I sat down and took my blood pressure twice before purchasing some sparkling water and calling the nurse again.

Since my blood pressure came back normal, the nurse said she wasn’t too concerned and advised me to better monitor my water intake since the cramping was likely due to dehydration, so I’ve been hydrating like crazy.

She encouraged me to call her back if cramping continued or worsened but I am already feeling much, much better today.

As I typed everything out to share with you guys this morning, I realize freaking out over light cramping may seem trivial, but I really was scared and felt very grateful for the ability to call a triage nurse who was so kind and knowledgeable and wish I just would’ve reached out to my OB/GYN sooner. I’m not sure why I let myself wait so long – probably because my cramping was rather light – but I guess part of me felt like I was being an overly paranoid pregnant woman since I have experienced more worry and anxiety during pregnancy than I ever thought I would.

Up until yesterday, part of me felt attached to our little guy, but part of me also struggled with really feeling *connected* to the baby boy growing in my belly. When I feel him move, there is nothing better and I am dying to meet him, but I definitely did not feel an instant bond or real connection to the baby growing inside me until yesterday.

I think it took feeling scared and overly paranoid for me to realize that I care so, so deeply for our baby boy. I want him to be safe in my belly. I want him to grow big and strong and enter this world as a healthy little newborn when the time is right for us to meet him. In looking for the silver lining in all of the anxiety I felt yesterday, I realized something rather huge: I am already *falling in love* with our little one. We may not have the connection I’m sure will come when I meet our baby and get to know him, but we have *something*. And it’s big and it’s powerful and it’s amazing.

**Question of the Morning**

- For the mamas out there: Did you ever struggle with excessive worry or anxiety during pregnancy? Was there anything you did that helped ease your mind and concerns?

**Content from Blog #6**

Sometimes, I forget how to breathe. It seems so simple–an innate ability we are all born with–but when thoughts and fears are rushing through my head at all hours of the day without pause or relief, it’s hard to remind myself how to breathe.

Muscles tighten, shoulders hunch in natural bodily defense, head pounds with the newest version of a migraine from hell. My hands and fingers twitch and clench and unclench like a heartbeat:twitch…open…close…twitch…open…close…

I dread speaking to people, even people with whom I’m (for the most part) comfortable around. Fearing I’ll say the wrong thing or be unable to follow the conversation and give a timely/appropriate response because I’m so focused/worried about how I’m standing, sitting, breathing, and listening, I tense up. And if I do dare to respond I ponder my response for days afterward, debating if it was an appropriate response or one I need to kick myself for.

People commenting on my appearance has always dredged up self-loathing and body insecurities I have had from childhood due to verbal abuse from my peers. Surely they don’t actually think I’m pretty and that my shirt is flattering. Surely my new hairstyle isn’t as attractive as they say. Surely they are lying to me to build me up and then talk about how ugly and overweight I look behind my back.

Some days, I don’t want to wake up at four in the morning to get ready for an eight-hour shift.

Some days, I can’t. I can’t physically dredge up the strength and walls of support I need to be around people all day.

Some days, I’m too depressed to even do my hair or put on clean pants. Makeup is easier; it makes me feel better about myself, makes me believe I am beautiful even though I don’t see it.

Some days, I can barely make it through work without wanting to scream at everyone to just stay away from me.

Some days, I cannot function as a human being.

It has been said that depression is like a cancer: sometimes, there’s no “getting through it.” There is no “giving it to God” as my childhood faith would have me believe. I want to believe that it can all go away with one simple prayer to my God—but I have tried, again and again, and it’s still here. I still carry it with me. Maybe some days are easier than others, and some days praying does help, but not every day. Some days, it just doesn’t happen.

Now that I am pregnant, these emotions are two-fold.

Blame it on pregnancy hormones or personal difficulties, but I’m more terrified than ever to be around people. Why? Because nobody has any boundaries when it comes to pregnant women. I will never understand why the mere sighting of a woman’s beautiful, growing belly will cause people to completely throw out their manners in less than two seconds.

The constant questions about my child’s gender, name, nursery theme, baby shower, which hospital I will deliver in, whether I will have an epidural or go “natural,” who I will have in the delivery room—they attack a young mom like me, like a medieval army would attack an unarmed city. Swiftly and without mercy.

And the judgments that follow these questions when I tell these nosy people what they want to know…talk about a rush of anxiety that morphs immediately into a state of depression. I can’t do anything right, apparently.

I know, with the little bit of maternal instinct I’m growing into, that I want to get better for my unborn daughter. I want—need—to go to counseling or therapy or a support group. Anything that will enable me to get through those days where I feel like a complete failure at life. I have to be better for her, that way she will grow up to be far healthier than I am.

I want her to be better than me.

That is not to say that I’m a lesser person for the issues I face, but I don’t want her to have to feel the way that I do. Ever. I love her far too much for that.

Yes, I deal with anxiety and depression. Yes, I am a younger mom. But no, I will not give in so easily to these struggles. I will fight to breathe. Always.

For her.

**Content from Blog #7**

My first pregnancy I was blissfully unaware of what pregnancy and childbirth were like, and honestly I wish that I could get a bit of that naiveté back. Every bit of those nine months felt new and exciting and fun. I loved learning about everything my body was doing and how my son was growing. My birth was a little scary at the end but with the techniques I learned in my Bradley classes, I was able to achieve my goal of an unmedicated birth.

The second baby was conceived quite by chance shortly before my first son turned a year old. The anxiety kicked in and I felt more isolated from my husband because of the demands of his job and his inability to well, care.

My entire pregnancy I hoped and prayed that the baby would be breech so I had an excuse to not go through an unmedicated vaginal birth again. My husband assured me that I would feel disappointed in myself and the experience if I “wussed out” and agreed reluctantly to help me re-study our Bradley book. Every time we sat down to go over it, I would have a rush of fear and start to cry or get angry and decide I was done for the evening. I just didn’t want to think about what the process was going to do to me again.

At some point in the last ten weeks of that pregnancy, I decided to be “tough” and “suck it up,” telling myself that having unmedicated births were the greatest gifts I could give my children on their birth days. Also I had decided this was our last baby, so I just had to do it this one last time.

The labor went quicker. I was strong and determined and confident. I kept my wits the whole time (well transition was the usual craziness, but I held out) and experienced another unmedicated birth. This time, a nine pound, one ounce screamer. And then the room fell silent.

I was holding my baby and didn’t notice much but wasn’t allowed to sit up, and then all I really remember was a lot of fundal massage and cramping and code words. Honestly, I felt like crap and couldn’t walk and was incredibly dizzy. Later that day a nurse informed me that I had hemorrhaged and they were about to give me a transfusion but I stabilized.

The OB who delivered my baby said something about me being past the point of blood loss that “we worry about losing the mother.” They then told me that the severe cramping was because I had been given Pitocin through my hep lok. They kept me an extra night for in hospital care for GBS+ reasons which I disagreed about and then guilted me into a heel stick for my baby because he was “big” and they wanted to check for diabetes.

Where was my husband for ALL of this? You tell me. I was ALONE. However, I knew that at least I would never, ever have to go through this again.

Fast forward exactly three years later and we are stunned by the conception of another baby who will be here in less than 13 weeks. I was in a funk for two weeks, and it took me three months to get back to reality and my responsibilities with my business and mothering.

Here I am, now, in some ways getting my mojo back and in others an absolute mess. I try to do what I can when I can in different aspects of my life to maintain order, but there is a lurking emotional monster somewhere near me at all times.

I have broken down now in two midwife appointments and cry whenever I think about having to do that again. I have been diagnosed with PTSD and clearly I have anxiety and depression which I have never experienced in my lifetime. I fear that I may die next time, that I may bleed out, tear horribly as I did with the first two, or just have something dangerous or scary happen. It takes up a good deal of space in my daily life. So what do I do about this?

I have answers from everyone and suggestions and loving advice but all I can think about is this: I am not telling anyone what I really want. What I really want is control. I want to have a birth I am happy about not because I “did it for the baby” or did it to make my husband proud or did it because I felt I had to after being given a sense of control from a child birth program. I want to feel I had my best outcome in a birth because I got what I needed to have closure. (I will be getting some form of permanent birth control after this baby, as will my husband.)

I NEVER in a million years thought I would say this, but I am seriously considering a c-section. Do I care if anyone judges me in this? Honestly, I did until the anxiety took over my life. I don’t want to let the anxiety in any more than I have. I see this as a chance to heal, and I don’t think I would have been forced to deal with all of the bottled up stress from my first two births if this third baby hadn’t come along.

I want to heal from this and I want to feel like I can close this chapter in my life and let it all go. I also want to make my body a safe place for this child while she is still there and right now, I know it is not. I can’t go on hoping to have the decision made for me and suffer like this all the way to the end and then come up with some last minute decision about how I will birth.

I’m still working this out in my head and will have a discussion with my midwife at the next appointment so I definitely don’t have concrete plans for anything yet, but I feel better knowing that I am finally taking full ownership of this and not allowing myself to feel pressured in any direction.

If I could say anything to anyone who utters one word to a pregnant or postpartum mom, I would urge them to take consideration for the mother and her well-being and not spend so much energy on the baby. Let her shower, rest, do the chores for her, hold the baby for her while she eats dinner, and let her cry to you if she hurts.

But most of all tell her it’s okay and it’s normal to feel whatever she does or doesn’t feel. Let her be in charge and own the entire experience and not feel like she has to put on a brave face for you. I wish I had been more vulnerable after those first two pregnancies so that I could be more open for this one.

**Content from Blog #8**

Because I’ve lived so long with anxiety, there were lots of things I figured I’d never, ever do — having children was one of them. In fact, in my 20s, before I’d managed to stumble on the combination of drugs and exercise that allows me to be as sane as I can expect to be, I was so sure I’d never give birth that I got a tattoo of a giant koi fish extending from one end of my abdomen to the other.

Seven years later I found myself hugely pregnant —the koi fish had expanded to a koi whale, stretched out into a giant, fleshy billboard advertising the grand impulsivity of my youth. On darker days, it was a villainous rebuke. Its growing eye stared back at me in the mirror.

At practically the very moment I found I was host to a living being, I realized that that living being could stop living.

To be clear, I did choose to become pregnant, and when I did I found myself in a very ironic situation: my decision to have a child was the result of a sense of stability I enjoyed due to taking an array of anti-anxiety medications that I would be strongly advised by most doctors to stop taking now that I was pregnant.

It was also my medicated, logical brain that reasoned that the possible side effects of the drugs on the baby would be even greater than the anxious thoughts and behaviors that would flood back in when I stopped taking the pills. I can do it! I told myself.

So I stopped taking the pills. And this is what happened.

My pregnancy was one long, nonfunctional funk, in which I oscillated between the couch and the bed and seldom removed my favorite pair of stained elastic-waist pajama pants. If I had to make a pie chart of my activity during pregnancy, the two largest sections would be “crying” and “apathetically watching ‘Law & Order’ reruns.” I worried that upon birth my child would not recognize my voice or that of my husband but only the gestational background noise she’d heard the most — the sound of a gavel slamming onto wood.

Pregnancy without drugs made it impossible not to constantly entertain the giant, looming questions of mortality all throughout the day. Few things can make one focus on death more than the life-giving process. At practically the very moment I found I was host to a living being, I realized that that living being could stop living. I remembered, that I, too was a living being who could stop living. Whenever I allowed myself the slightest glimpse at the new potential zenith of loss made possible by this gift of a child, I became paralyzed.

The specter of doubt in my head was a convincing one — it took on the form of a wizened voice that brought to mind an elderly philosophy professor in a sweater vest sitting in a rocking chair and thoughtfully puffing away at a pipe. “Just look at you; look at your life!” the voice said. “How exactly would someone like you MacGyver a human baby from the ether of sarcasm, Eggo waffles, and dog hair that is your existence?” It’s true, I thought. It’s enough of a triumph for me to remember to turn off the coffee maker each morning that I’m literally proud when I do it. Did I think I’d actually somehow managed to conjure up — and nurture — another person?

This, of course, spawned worries for me that the pregnancy wasn’t proceeding correctly. For example, the “nesting” energy I’d been told about did not kick in and make me want to clean things. A dust ball behind our bedroom door slowly grew to the size of a small cantaloupe. Instead of removing it I manufactured an odd link between the growing dust ball and my growing fetus: I did not pick it up or throw it away for fear that tossing the dust ball would cause me to miscarry. When it grew so large that our dog began to regard it with suspicion — sitting several feet away, trembling, emitting a low growl — I finally compromised by vacuuming it up but not emptying the vacuum into the trash: it was still safe, I assured myself. Encapsulated and going nowhere. I could toss it after I gave birth.

On the bright side, pregnancy actually decreased my anxiety in certain areas — mainly relative to physical appearance. Leaving the house without changing into actual clothing, for instance, would’ve made unpregnant me feel self-conscious, but pregnant me never gave it a second thought. It made going through the McDonald’s drive-thru while wearing a bathrobe at 3:46 p.m. on a weekday feel seven percent less embarrassing than it had before. Ditto for pumping gas in oversized tropical bird patterned pajama pants. When someone at a stoplight recently used their phone to take a picture of me drinking from the mouth of a 2-liter bottle in my car, I was blissfully unaffected. In fact, I had to actively rein in my apathy at others’ judgment of my sloth.

Amazingly, my baby was born without a hitch. And I’ve begun to resume the taking of my beloved anti-anxiety drugs.

But now that I am a mother, I am at the threshold of a whole new set of anxieties I haven’t even allowed myself to contemplate. I’m about to enter a club where increased anxiety is the norm even for people with regular brains — so where will that leave me? When I do allow myself to think about what’s beyond the cliff, it’s the worry that I won’t be able to handle the worry: that no amount of medication, cathartic yoga, or omega-3-enriched, low-preservative-diet food could ever possibly be enough to handle it. I worry about the games and tricks my brain will play on me in its misguided attempt to try to help me cope with the hurricane of what-ifs. But if there’s one thing I’ve practiced to date in my life, it’s anxiety. If anything has prepared me even slightly for what’s about to come, maybe it’s that: maybe my life-long nemesis was just a gateway to motherhood all along.

**Content from Blog #9**

Guilty. The answer is, surprisingly: guilty. At least, that’s the answer I have for you at this moment.

Happy. That’s the answer most of the time. I am so happy. I am so happy that I could cry with gratitude. And I have.

Relieved. I am so relieved that this is a time of respite. No turmoil. Just calm and love.

I don’t know why. I don’t know how. But my anxiety baseline *plummeted* when I became pregnant. I’m not talking about zero. I’m not even talking about person-with-no-anxiety-disorder levels. After all, I’m still anxious about taking the baby I nanny for a walk. (I have no idea why. It’s not rational.) And I have no excuse for not showering even every other day. (Just be grateful I don’t have strong body odor and move on. It’s not rational.)

But I’m not afraid of laundry.

I’m not afraid of leaving the house on time for work or appointments.

I’m not afraid of doctors, not even my psychiatrist.

I’m not afraid of my meds.

I’m not afraid of cooking.

I’m not afraid of doing dishes.

I’m not afraid of cleaning.

I am excited about my upcoming project of putting my regular person clothing in Rubbermaid tubs and clearing out my fabulous Ikea wardrobe for just maternity wear.

I’m excited about organizing our things while we move.

I’m excited to move into the bigger, nicer apartment across the hall.

I’m not afraid of money. I can look at the bank accounts, the bills, the upcoming bills, and no matter what, my heart rate doesn’t even rise.

I’m not afraid to go to therapy or to cancel when I feel sick.

I’m not afraid to rest when I’m sick or just plain need to rest.

If you had given me this list just a year ago, maybe even just this January, I would have said, “That’s not me. It will never be me.” The doctors told me that there’s always a chance that hormones during pregnancy can help you feel happy, not make you anxious or cranky. My mother and sister were both pretty serene pregnant women. But then, they don’t deal with the kind of anxiety I’ve faced, so that always made sense to me. I couldn’t take their experience as a predictor of my own. I prepared for the worst. I braced myself. I warned my husband to brace himself. And then, the test turned positive…

Since then, the world has just settled into place. It has stopped spinning or tilting at random. I literally smell flowers and the rain more intensely (that’s a pregnancy symptom–a super sense of smell). I haven’t felt panic in, oh, about 14 weeks. I know that I still need the medication–if I forget a dose, the anxiety creeps back up. But I am serene in my knowledge that I have made the best choices I could make for me and for my family.

And now, I feel guilty. All that preparation, this entire blog, and I turn out to be the poster child for a happy pregnancy? Ok, not quite the poster child (there are those meds, after all). But seriously, I am happier pregnant than I was before. I am happy. What about all the women I’ve found through this blog who relate to me? Will you still relate to me if my moods remain even? Will you resent me for reacting so well to pregnancy, which has been such a scary experience for some of you? *I* resent me for reacting so well to something that, according to… who? that’s a good question… according to someone, was supposed to make me an emotional wreck and unpredictable hormonal nightmare.

I was afraid that I would run out of things to write about. I’m glad that I haven’t. I am so glad that I have been given this reprieve. I thank God every day. I do not take this drop in anxiety for granted, not even for a minute. Maybe it will give the book I want to write the kind of happy ending readers love. But who am I without my excuses? Without my disorders? It’s really pretty exciting to find out. Go ahead, strip them from me. I’ll find a new identity. Happily. I still have a strong voice. I am still *me*. And that is just… *weird.* It’s completely bizarre. I don’t understand it.

But that’s the thing about gifts from God: I don’t need to understand. I take them and say, “Thank You.”

Bug, at 14 weeks, you are apparently the size of a lemon. But you are sweet as pie! Thank you for all the sweetness you bring to my life, every day.

**Content from Blog #10**

In my 20 week pregnancy update I mentioned I had been dealing with some pregnancy anxiety the week prior. Thankfully it has been much better this past week, but I'm sure I will have bouts of it again over the next 19 weeks!

I really was having more of an issue dealing with mini panic attacks than anxiety (which is more pervasive and ongoing than the acute issues I was having.) I immediately flashed back to when I was in middle school and first got my braces put on. I remember crying in the shower when I got home knowing that for the next two years I wouldn't be able to take them off my teeth. My teeth and jaw were throbbing, I hated how they looked, and I had zero control over it. My pregnancy was starting to feel similar.

I would see photos of women nearing the end of their pregnancy, see how giant and stretched out their stomachs were, and start to panic. I was already having such a horrible time breathing I couldn't imagine getting *so much bigger* and being able to function. Then I'd start to imagine labor and how painful it will be, and what life with a constantly crying newborn would be like, and suddenly I felt like there was a 100 pound weight on my chest.

As with most of my panic attacks I think the trigger was physiological; my irregular breathing. Once I started catching on to that early I was able to stop the panic attacks entirely. During pregnancy I've avoided almost all of the things I used previously to calm my anxiety; teas, essential oils and vigorous exercise. It's crazy but the solution to my problem was so simple! {Skip to "how to manage it?" below to see what worked for me.}

**What is pregnancy anxiety?**

Anxiety that is experienced while pregnant. Some research has found that up to 33% of women experience clinical depression or an anxiety disorder during their pregnancy, yet fewer than 20% seek treatment.

**Who is prone to get it?**

- Women with a history of anxiety or depression
- Being a young mother (under 20)
- History of premenstrual dysphoric disorder
- History of miscarriage/s
- Pregnancy complications
- Poor social support
- Financial difficulties
- Marriage/relationship problems
- Low Income
- Having more than 3 children

*Really - who doesn't have at least 1 or 2 of those risk factors?*

**What are the symptoms?**

- Excessive worry
- Irritability
- Muscle tension/aches
- Insomnia
- Fatigue
- Feeling restless
- Inability to concentrate
- Panic attacks (may be a separate diagnosis of panic disorder)

**What are the risks?**

- Low birthweight
- Premature birth
- Low APGAR score (test of how healthy the baby is immediately after delivery)
- Respiratory distress and jitteriness

**How can you manage it?**

- The #1 thing that helped me with the mini panic attacks was taking 10 deep breaths in. That's it. In the midst of a anxiety/panic attack it's about the only thing I could manage. I'd inhale while counting to 5, and exhale counting to 5. Honestly by the 3rd or 4th breath I'd already feel better. I also keep some lavender essential oil on the end table now to take a whiff of if the breathing doesn't help.
- Eat well and avoid large amounts of sugar and caffeine. Caffeine is a *huge* trigger for anxiety!
- Exercise daily! Even if it's just a 20 minute walk. Move. Preferably outside in the sunshine and fresh air.
- Seek professional help. I'm still on the hunt for a good therapist in the area that specializes in cognitive behavioral training.
- Make sure you are getting enough Omega-3's (I love this one [hyperlink removed to de-identify] for vegetarians.)
- Embrace self help books. I love: Anxiety and Phobia Workbook, Mindful Way through Depression, Worry, The Now Effect and Full Catastophe Living.

**Content from Blog #11**

Before I get into the ways I de-stress, I want to tell you a story about what happened when we found out we were expecting Baby Bird. It may not be the story you imagined and I've been quite anxious about sharing it but I hope it helps illustrate how stress and anxiety hit me at the weirdest moments!

**Stress happens to even the happiest people**

Baby Bird was planned, or rather wished for, hoped for, dreamed of, loved already.

We knew what we were doing. We wanted a baby, a child, a family, a new chapter in our lives. And we were very lucky. It happened naturally and quickly. When I told my partner [Name de-identified] that I was pregnant, the news was greeted with the widest smile he is capable of producing (which is remarkably wide; he has a very large mouth) and a few jokes about how the baby would be as hairy as him so I better prepare myself for some serious inner carpet burn (!).

However, my very initial reaction was less smiley.

When I first found out I was pregnant - a few hours before I broke the news to him - I was sat alone in the downstairs toilet of our old apartment staring at a barely-there second line indicating what might be.  I remember staring at myself in the mirror, my eyes as wide as the moon and an undeniable fear making my skin sickly pale. My first reaction was to say a certain four letter word multiple times and by the time I'd grown a bit bored of how that word sounded, I let myself cry. A lot. I realised I was in a deep, dark, uncomfortable shock and this made me cry more. I wanted this, didn't I? Yes. So why did I feel so confused, scared and uncertain about everything?!

It was early on a Sunday morning and through the tears I realised I should take some time to get my head round the new information by myself before rushing to tell [Name de-identified] what should be happy news, but was about to be relayed to him by an ugly, frightened version of the woman he's trusted to carry his child.

I finally left the toilet, the test in my hand - that second line stronger, bolder by now - and sat down on the sofa in our living room. I took a series of deep breaths. Still the tears came, and the fear, and the anxiety, and a few more of those four letter words. It got to a point where I couldn't breathe properly and that's when I knew I had to stop. So I breathed in deeply and slowly. I closed my eyes and told myself I didn't have to do anything at that moment but calm down. And eventually, that's what I did.

A few hours later, upstairs with [Name de-identified], who was still grinning like the Cheshire Cat on stimulants, I too was smiling. I was smiling so much it hurt. And together we have smiled our way through much of my pregnancy.

However - yes another one - despite wanting this, loving this, enjoying this, I have also experienced an increase in anxiety and unpredictable amounts of nerves. I can blame this on hormones to a point, but also I know well that I'm a very emotional person, and what is happening to my body and my life has meant that I've become more prone to small bouts of stress over the last seven months. I was especially anxious during my first trimester which is why I started to explore yoga, meditation and mindfulness like it was my job.

Maybe I will write more about these explorations in due course, but for now, I want to share five simple things that have helped me more than I would have imagined to quickly reduce a speeding heart-beat, to slow a hot rush of perspiration, and to bring me back to a calmer, clearer place to think and act, or to just move past the source of stress (which I've found is more likely to be the necessary course of action!)

So I hope these five quick tips for dealing with a sudden (or not!) bout of stress or anxiety help you too. Anyone can do them and while they may not solve all your problems in five minutes, they will help you feel better.

**Five things that will help you de-stress in less than five minutes**

**1. Breathe**

For as long as I can remember, "taking a deep breath" has been advice that has been dished out to me (by my wise parents, a teacher, medical staff and also [Name de-identified]), but it wasn't until the last year that I actually considered why we are told to breathe deeply when we're panicking, stressing or upset.

**If you slow your breathing, you slow you're heart rate.**A fast heart rate is one of the most indicative signs of stress and anxiety, so you're effectively nipping one of the biggest symptoms in the bud before it becomes out of control and dangerous. Slow, deep breathing also gets more oxygen to your muscles (they like that!) which will in turn relax them, so your body doesn't feel like coiled spring as much.

**One of the best ways to do this actually isn't by taking in a deep breath, it's by letting out a deep breath.** A long exhalation activates the vagus nerve, part of the parasympathetic nervous system, which is responsible for counteracting or calming the fight or flight nerve system, the sympathetic nervous system, which interestingly enough is activated when you inhale. **An active vagus nerve (i.e. a long exhalation) helps you "rest and digest"** rather than assemble a load of adrenaline and try to attack the stress or problem causing it. Of course, I didn't know all this off the top of my head, I did quite a bit of research and this article goes into more detail about "vagal tone" if you're interested.

If you extend your exhalation you not only help keep the vagus nerve active, but **you also extend the amount of time you "live in a moment" thus slowing down your sense of time and urgency**. I don't really have much science to back that last one up; that's just something I've learned through practising deeper breathing.

**2. Stretch**

Before I started going to pregnancy yoga (at the brilliant [Name and location de-identified]) I was already aware of the benefits of stretching. I'd love to say it was from my fictitious previous career as a semi-professional athlete or even from my very real and painful years of running recreationally, but no, actually, it was from watching the neighbourhood cat who considered our old apartment his second home.

The day had long turned sour. I'd gone over the amount of time I'd allocated for a job and still hadn't finished. I had unpaid invoices to chase. The kitchen needed cleaning but I didn't have time to do it, and it had been days since I'd worked on London Eyes, the book I was then editing. The cat was sat to my left, resting on a pillow in the window seat there. After sleeping for an hour or so, he stood up and proceeded to go through a five minute routine of stretching. He arched his back; he pushed out his front paws, one at a time; he jumped down from his seat and before walking away, stretched out his back legs too.

**It struck me that if a cat stretches instinctively, and regularly does so, and he doesn't have a physio or a personal trainer nagging at him to do so, then maybe, there really are benefits to stretching regularly.** I then remembered how good stretching feels when it's not at the end of a workout or when I feel "I have to". I immediately stood up and stretched out all of my limbs from the tips of my fingers to my toes. It took little more than ten minutes, but I felt like a new person. You don't have to know yoga to stretch; you just need to elongate your limbs to remind your muscles that and let a little more air into your lung

If you're prone to injuries of any sort, I'd recommend getting a professional to advise you on the best way to stretch.

**3. Yawn.**

I'm always amazed by how contagious yawning is - apparently, even reading this paragraph will probably want to make you yawn. But little did I know that it also has real benefits, many of which can help you at times of stress or anxiety.

When you yawn you aren't just publicly putting someone's topic of conversation down or telling your partner that you're too tired to do the dishes, you're actually engaging your brain in an important and beneficial activity. The act of yawning - forced or otherwise - activates part of the brain called the "precuneus" which plays an important role in self-reflection. **Many neuroscientists believe that yawning is a sort of "restart" for your consciousness of what is going on around you at that time** and my yoga teacher always says that yawning in her classes is a real compliment for her as it shows we're relaxed and feeling calm. I've also read that many therapists use yawning as a technique to reduce anxiety and tension, be it physical or emotional. Who knew yawning was so effective at dealing with stress?!

And for the record, I yawned no fewer than eleven times writing that.

**4. Put your feet up.**

As an 8-month pregnant woman I'm a huge fan of putting my feet up. Heck, I've always been a fan of it.

While the benefits of raising my feet higher than the rest of my body during pregnancy have been explained to me at great length - reduce swelling, improve circulation, rest is good for mother and baby - I didn't expect it to actually *feel* so good. It may just be me, but **the simple act of sitting or lying down and then raising my feet up on a couple of cushions or on a chair if I'm lying on the floor seems to reduce the weight on my body and brain**. I suppose much of it has to do with how it improves your blood circulation, which is the same kind of benefit a massage will offer you. [Name de-identified] actually swears by having his feet raised at all times when he's working at his desk and there's some evidence to support this. Personally I just look forward to that 30 - 60 minutes at the end of the day when I can raise my feet and yawn my head off... But **I have also been known to take a break from a stressful activity and lie on the floor with my legs on my desk chair just to feel a lightness and new perspective return to my overworking mind**. (For those of you who need more convincing or direction, these yoga poses that focus on elevating the feet and legs on a wall are supposed to energise as well as reduce stress.)

**5. Laugh.**

They say laughter is the best medicine and maybe like the "take a deep breath" advice and that stretch-happy cat, we too quickly overlook exactly why this is the case. I got to researching this too and found this article which explains that **laughter actually has "all the reciprocal, or opposite, effects of stress"**.

Unlike yawning (and there I go again with number twelve!), I don't find it easy to force laughter and reap the benefits, so I would highly recommend having something at hand to get your laughing muscles exercised. I have a handful of YouTube videos favourited, a collection of photos on my phone and a very generous boyfriend who has mastered a very silly dance that always gets me giggling. I admit that this is the hardest one to consider when you're in the midst of an "the end of the world is nigh" anxiety attack or you've had a particularly stressful day, and if you need more than five minutes to watch an episode of your favourite sitcom or to read a chapter from a funny book (or you could try these extracts of my stories that are almost amusing) then do it. You're not skiving or being lazy; you're fighting stress.

Just remember to put your feet up and breathe deeply as you do....

What things do you do to try and keep stress or anxiety at bay? I'd love to find out.

**Content from Blog #12**

A few months ago during the beginning of one our prenatal yoga community classes, a new student caught my attention. I am not sure what it was about her that I focused on, there was nothing peculiar about her that spoke out to me, just an air of unease. During circle time, she introduced herself and said the usual; her name, how far along she was and whatever ache or pain she wanted to address in class that day. Nothing unusual was disclosed. She quietly moved through class and seemed to enjoy the yoga and the community of pregnant moms around her.

After class, she approached me and asked if I have a moment to talk. “YES!”, I said-and was delighted to have an opportunity to check in with her. She introduced herself to me and told me her name is [Name de-identified] and wanted my opinion on a pregnancy related matter. Within moments, tears started to well up in her eyes. She shared that her doctors wanted her to go back on a medication for anxiety and asked if I had known any other women who had taken a medication during pregnancy. I shared that I had in fact known two women who had taken medication during pregnancy, and validated her decision to pursue this path with her doctors. We talked for a while and by the end of our conversion, [Name de-identified] seemed to have lifted a heavy weight off her shoulders.

**[Name de-identified] Story**

I didn’t really put this together until more recently, but I had been on psychotropic medication for about 15 years. And the psychiatrist I was seeing, while he was supportive he was not picking up on important cues from me regarding how I was doing once I became pregnant. In April- my husband and I made the decision to have a baby and I would come off my medication very slowly in order to minimize the withdrawal effects. I was on Effexor for a total of seven years, an SSNRI (SerotoninAnorepinephrine reuptake inhibitor) as opposed to SSRI (Selective serotonin reuptake inhibitor). SSNRI’s are known to be effective, but Effexor in particular is a bear to come off. It is also not recommended to take during pregnancy since the drug passes through the placenta and affects the baby. The withdrawal effects for the baby are challenging.

I could have transitioned to an SSRI, but I thought “I can do this, I don’t need anything right now.” So I did not transition to a new medication I just came off my current meds. By the end of July, I had stopped taking Effexor and immediately in August I became pregnant. My psychiatrist didn’t have any strong opinion about this. In hindsight, I feel like he was not seeing me and not really looking at what was happening. He missed some big warning signs. Before I was pregnant, I never once texted or called in a frantic state. In September I began frantically reaching out to him for guidance and support and- he just didn’t pick up on the cues that I that was struggling During this time, I was trying to be proactive and took a holistic approach, I was doing acupuncture, taking fish oil- trying to do all these supplemental things to help.

Within two months of coming off Effexor I was a mess and in the middle of my first trimester. I felt so ill-equipped to cope with normal biological pregnancy symptoms like nausea and fatigue. My serotonin had been so depleted that I didn’t have a supply to support me during the huge hormonal shifts associated with pregnancy. I had heart palpitations, difficulty sleeping, racing obsessive thoughts. It was so difficult coping with these physical symptoms of pregnancy, I was crying all the time and completely overwhelmed. I thought this was the worst thing that ever happened to me. I truly believed I had made a mistake. I had no business getting pregnant. I found it particularly difficult being with other pregnant women. It was hard to get real and relate with what they were feeling. Everyone would talk about feeling so great. I was thinking “Why don’t I feel fine?” I felt isolated when everyone else was having such great pregnancy experience and I was suffering.

A turning point came at my 3rd appointment with my OB. During my first visit I discussed my history of depression and anxiety with my doctor and she said I could stay on my medication. At the time it was early September and I felt like I could do this on my own. By my November visit, A however, it was quite clear to everyone but me that I was not coping. My OB called me out on my shit. She was the most direct provider I had ever met and clearly told me, “You need to go back on your medication. Even though other women have these physical symptoms, they are not affected the way are you. You are suffering and you don’t need to be. Your homework is to get back on your meds.”

I went to a reproductive psychiatric specialist at The Women’s Program at [Name of University de-identified]. I was immediately told that, for someone who has been on medication for a long time- pregnancy is exactly the WRONG time to come off it. We know the results of anxiety on the baby, increased risk of developing preeclampsia, premature birth and low birth weight. But what they have not seen is any consistent evidence of danger to the baby associated with the use of SSRIs during pregnancy. I felt reassured when I learned, in Europe there are years and years of data for women taking SSRIs during pregnancy which has shown a clear lack of data on the development of malformations with women on these medications. In other words, while it is unethical to do a case control study with pregnant women, there is substantial data that indicates there is nothing to find.

Even though intellectually I knew it was vitally important for me to go back on medication, it was hard to emotionally accept. I felt at times even my husband was judging me. After many very intense conversations, he started to see that it was more harmful to NOT be on something, than the risks to the pregnancy to be on something. I don’t tell people that I am on medication and those I have told are very few but supportive. I don’t believe medication during pregnancy is publicly accepted. Everyone is so hypertensive, like no one wants to take even an Advil! – I feel there is a judgmental mindset to taking anything. That all natural approach does a huge disservice to pregnant women, and an even bigger disservice to those who need medications. There are risks and benefits to everything. I really believe- Happy mom = happy baby! If mom is suffering that is never good. People are so fearful of hurting the baby they may overlook the true need for some non-natural approaches. As a society we need to remember, if we take care of the mother, we are helping the baby.

I ended up going on Zoloft and an increased prescription for folic acid and larger dose of the fish. The belief in adding these supplements is that it is adds additional support for depression and anxiety which helps to keep the dosage low. Back on my medication, the waves are not as high and low. Now I can cope with these waves. I am not having as much of the heart palpations, and the racing thoughts are more manageable. That is really the distinction- it is manageable. Issues don’t go away- but I can manage it better.

I also try to remind myself, not every day has to be a great. I had put this tremendous pressure on myself to enjoy everything and sometimes that is not the case. It is ok to not like pregnancy. You don’t have to be this Hollywood image of the glowing happy pregnant woman. Before pregnancy, I thought I would be super happy and be on a high all the time. I was pretty shocked that it has been such a struggle. But now I know I am going to be ok and I look forward to meeting my baby.

**Finding Information and Help**

Here is the website for Harvard’s Mass General Hospitals Center for Women’s Mental Health. They have specific information on breastfeeding and medications, and pregnancy and SSRIs. This particular article is Psychiatric Disorders During Pregnancy

Finally, this is the link to Harvard’s Pregnancy Library. This offers tons of helpful resources.

**Content from Blog #13**

Anxiety in pregnancy isn’t often talked about.

From the positive test to the moment that you hold your new baby in your arms, pregnancy is expected to be a time of excitement and joy.

Yet for many women it is a time of great worry and anxiety.

In recent research from the IONA test, at least one in five women report experiencing extreme worry and many mums-to-be admit to regularly losing sleep over concerns for their unborn child.

Sadly, this research echoes my own experiences.

I am 35 weeks pregnant. And this pregnancy, my sixth, has been filled with anxiety, stress and fear.

It has been a struggle and a fight.

We are lucky enough to have a beautiful daughter but tragically we’ve also had four miscarriages.

We are now less than five weeks away from meeting our baby boy.

I wish I could say that this has been the wonderful pregnancy I desperately wanted. But that is not true.

My anxiety levels rocketed the moment that I discovered that I was pregnant.

During the first trimester, I could only see one possible ending.

An ending that was sadly all too familiar – the loss of another baby.

The past eight months have been filled with fear.

I have been terrified that every twinge or pain was a sign of miscarriage.

I have been constantly worried about bleeding and I have questioned every pregnancy symptom that I’ve experienced.

I’ve cried every day and every evening my poor husband has had to listen to me talk over the same fears and concerns.

Every night I have dreamt of losing the baby.

And I have woken terrified, hugging my belly, believing that the dream was reality.

Even now I am filled with anxiety.

I cannot believe that we may soon meet our second child, a child that we thought we would never get to meet.

Pregnancy is amazing. I wish that every woman could appreciate this.

I wish that they could cherish their pregnancy, bond emotionally with their unborn baby and look to the future with excitement.

But instead it is often a time of heightened emotions, fear and worry.

Left to deal with the feelings alone and without support, it is unsurprising that many women are left feeling isolated, lonely and depressed.

My anxiety was at its height during the first trimester. And I know that I am not alone.

Many women struggle in the first few months of pregnancy.

Yet getting access to medical professionals during this time is almost impossible.

It is no longer necessary to report your pregnancy to your GP, you can register with a midwife online and you are only likely to have one appointment with a midwife before your 12 week scan.

This can all lead to unnecessary worry and fear that there may be something wrong with the baby.

However, these fears could be easily alleviated by providing pregnant woman with the opportunity to be able to talk about any worries that they may have during the crucial first trimester.

There should be greater access to midwives.

Women who are especially anxious should be referred for therapy.

And all women with high-risk pregnancies should be offered earlier scans or non-invasive prenatal screening tests to help put their mind at rest.

If your friend, family member or partner is suffering from anxiety in pregnancy then you will also want to know how you can help them.

It is important to remember that many women will not want to talk openly about their pregnancy until they have had their 12 week scan.

And this needs to be respected.

Even if you are certain that someone is pregnant, please don’t ask.

Instead, let them tell you their news when they feel ready.

After all our miscarriages, I felt that there was no need to hide this pregnancy.

However, before telling friends and family our news I asked them not to congratulate me.

Getting pregnant felt like one tiny step on a much longer journey.

During pregnancy, I have never felt entirely happy receiving presents for the baby.

If you wish to give your friend a gift during their pregnancy then consider buying a treat for the mum-to-be.

Pregnancy is a very private matter and every woman reacts to the news differently.

They may want to talk or they may not.

They may want to cry on your shoulder or they may want to try and forget their worries by pretending that nothing is wrong.

The main thing is to let them know that you are there for them.

If these worried and anxious women could be provided with more support and particularly during the first trimester then this could make a huge difference.

It could be the difference between a pregnancy of worry and fear or one of joy and excitement.

And all women should be allowed to experience a happy and anxiety-free pregnancy. I wish I had.

**Content from Blog #14**

I’ll never forget that moment. I sat in my doctor’s exam room, eagerly awaiting a first glimpse of my baby’s heartbeat. I imagined watching it, a tiny flicker on the screen, feeling instantly connected to the life inside me. I never imagined the doctor looking at me and saying, in his most clinical voice, “I’m sorry. There’s no heartbeat.”

That moment has stayed with me, seven years and two kids later. A miscarriage is a scar that never fully fades; no matter how much time goes by, a shadow of that loss always lingers. This time of year I revisit that loss, because every October 15 is Pregnancy and Infant Loss Remembrance Day. It’s a time to come out of the shadows, to let others know they’re not alone. And it’s a time to reflect — on dreams abruptly ended, on the pain of letting go.

For me, losing a pregnancy was difficult. But what was surprisingly harder, in some ways, was being pregnant again after this loss. All around me, friends were happily announcing their pregnancies, throwing showers, dreaming of their children’s future. I wanted to feel joyful about my pregnancy. But deep down, I was terrified. Between the constant fear of things I couldn’t control — and obsessing over those factors I thought I *could* — I was a wreck for nine months. There are many reasons why being pregnant after a miscarriage is different. Here are just a few.

**1. Fear of the unknown.** When I became pregnant with my daughter, I dreaded my first prenatal visit. Relief at seeing a heartbeat quickly turned to panic at learning my progesterone levels were low. As I filled the supplement prescription, I felt myself being submerged into an all-too-familiar nightmare. After each appointment, I waited to learn my “levels” like a prisoner waiting to hear her sentence. I felt trapped in a body that didn’t work, that couldn’t hold on to what mattered most. Even when I made it past the first trimester, my hesitations continued, shrouding every moment in a layer of anxiety and fear.

**2. Loneliness.** With my first pregnancy, we told our families right away. It was Christmas, and we were celebrating. I never dreamed I would be un-telling everyone a month later. With my next pregnancy, I was determined not to make the same mistake. We waited a long time before telling anyone, which made me feel protected, but also alone. My pregnancy became a secret to keep, instead of happy news to share. I held everything inside — exactly when I needed my friends and family the most.

**3. Guilt and self-doubt.** During this time I often questioned my decisions, worried that any lapse in judgment might end my pregnancy. I was nervous to lift chairs at a work event, scared to help a family member carry luggage up the stairs. (Positions I found myself in because I was too afraid to tell anyone I was pregnant.) When you’ve had a miscarriage, it’s easy to grab hold of the idea that by doing everything “right” you have some semblance of control — and, by doing something “wrong,” you’re to blame for the loss. It’s a heavy burden to bear.

**4. Morning sickness envy.** Most women dread morning sickness. I found myself longing for it. I craved the physical reassurance that things were “normal” inside me. I desperately wanted to know that my baby was there, growing and developing as expected. My lack of morning sickness felt like a punishment. Every moment I didn’t feel nauseous was a reminder of how little I understood what was happening inside of me — and how little I could control it.

**5. Fear of joy.** For a long time, I found myself prefacing every statement about my baby with “If we make it through the pregnancy” or “If the baby is born.” I was afraid to be excited, terrified of letting in too much joy. I was uncomfortable buying furniture for the nursery, shopping for onesies, or even thinking of names. Looking back, I wish I had let myself enjoy it more. But my wounds were still fresh. I didn’t want to indulge hopes that might lead to another heartbreak.

Sometime in my ninth month I began to relax, to feel hopeful that soon I’d hold my baby in my arms. I stopped saying “if” and started saying “when.” When my daughter was born, I knew she was the baby I was meant to have. But as blessed as I am with my children, when I hear of a friend’s miscarriage, I feel a pang in my heart. I’m glad that there’s a day to shine a light on this issue — for people to acknowledge and share their sadness about such a difficult subject. It was this openness — talking with others who’d been through it — that helped me get through those nine long months. Hopefully this openness will help others to know that there’s light — and even hope — beyond the darkness.

**Content from Blog #15**

“How are you feeling today?” The nurse asked me this past Wednesday, as she closed the door to the tiny exam room for my 16 week checkup.  Those words apparently were all I needed to hear to release the pent up anxiety hiding inside me behind a façade of calm and collectedness.  Alligator tears came pouring out of my eyes.  As I sobbed, “I said fine.”  This was obviously NOT the case.

I know anxiety is common, even normal during pregnancy. I remember having the typical concerns during my first pregnancy: *Will this baby be healthy?* *Is that food safe to eat?*  *Will I be a good mom?*  All these worries, and more, ran through my head on a daily basis, sometimes more than once a day.  I mean, it’s a BIG responsibility growing a new life inside you and it’s scary.

My fretfulness during this pregnancy has been 10 times worse as anxiety is now my constant companion.  I expected to feel more anxious with baby No. 2 after losing baby No. 1, but I never thought worry would be as persistent as it is.  Living with anxiety during a pregnancy after a loss is like walking on a tight rope for nine months, with no safety net below, just waiting in fear that I will slip and fall.

Even the littlest things can send me into a nervous spiral, an ache from round ligament pain, a cramp from constipation, and silence from the ultrasound tech, all of these increasing the already overwhelming amount of worries asking the question, “Will baby be OK?”  This past week one of those anxiety whirlwinds hit when I remembered that I had not heard back from my doctor about my most recent lab work to test for birth defects.  The nurse informed me that I should have the results back within twenty-four hours from my appointment.  As I thought about this sitting on the couch after work, I realized it was 5:30 p.m. on Friday!!!  My mind immediately spun in all directions conjuring up every negative possibility that could be WHY I didn’t hear back from the doctor on time.  “This must mean something is wrong.” My mind wouldn’t even comprehend that it could just be a clerical error, no; it decided to do fearful summersaults, over and over again until I was dizzy.

I sat on the couch in dread as there was no one to talk to, my husband wasn’t home yet, and the clinic was closed.  Not knowing what to do, I cried.  Tears of fear emptied out of me as I succumbed to being powerless and anxiety stricken.  Out of desperation I called the afterhours nurse’s line in hopes that someone there might have an answer.  Luckily, the kind angel on the other end of the line was able to calm my fears and reassure me that my test result came back negative.  Baby was fine.  I was fine.  Relief set in and I cried all over again.

Overwhelming anxiety can be a real concern if left untreated in pregnancy. I have come up with a list of ways to manage my anxiety for the next 6 months that work for me and that I have discussed with my doctor and therapist.  I share in hopes that you too might find a strategy below helpful, if you also struggle from anxiety after loss or just typical worries that come from creating a life for 9 months:

- **Practice Self-Care:** Going to bed early, giving myself permission to do less housework, and spending time relaxing with my husband and little dog.
- **Use the “What If Question” to My Advantage:** “*What if (insert negative thought here) happens?”*  Then I envision myself coming up with a solution or possible future action.  It usually ends with, “Even if the WORST happens, I can handle it.”
- **Avoid Google:** I know I do it.  As soon as there is a concern I turn to Google for help.  As if it’s a magic eight ball that will solve my problems.  When in reality, I usually walk away more fretful about my most recent worry than I did before.  I no longer Google concerns, I call the nurse.
- **Talk to a Supportive Loved One:** Talking to my husband, therapist, doctor, nurse, and other pregnant friends, has been extremely helpful in reducing my anxiety.  Surrounding myself with supportive loved ones has been some of the best medicine to help me through anxious moments of both pregnancies.

**Content from Blog #16**

*This is a post about anxiety and depression. It might be triggering for you if you’re currently struggling with your mental health. If you read this and it strikes a chord with you or you think you might need help – please see your midwife or GP or if you’re in [Location de-identified] call [Program name de-identified] on [Phone number de-identified]. This is just my journey, every one has a different journey. It’s scary to post this publicly so please consider that before you comment.*

I bought a book and I was scared of opening it. I don’t want to curl the pages. Read a book once and it’s read. I wanted to keep it perfect. Even when it meant I would miss out on the wonder in its pages.

…

When I woke the room was dark and silent. I reached over to touch my best friend and lover’s chest. I wanted to feel it rise and fall. I was scared I would not feel it.

….

My heart beat so fast I could feel the blood. I could hear it raging. I felt cold and tried to feel my feet on the ground to bring me back to the earth. But it pulled me down too far and everything went black.

…

I had a dream of a raging river. I filled my pockets with stones. It was romantic. They found my baby safe in a basket by the river. She had flowers in her hair.

…

When I was about six months pregnant with my second child I woke up one day and I couldn’t move. I thought I must have had a stroke or something. My husband asked me if I was OK and I burst into tears.

I’m not ok I said.

He said he knew. And he asked if we could call the midwife together. I was terrified. Telling our midwife I needed help was absolutely the scariest thing I’ve ever done. I was sure they would take me away or worse – take my children away. But I had made a promise to myself when we decided to try to start a family – I would be the best mother I could be. I would protect us by protecting myself. My midwife was amazing. Things happened quickly. I was put under the care of maternal mental health.

For me, in [Location de-identified], under this service – it saved us. I know this is not the case for everyone, and that in many places mental health support is under-resourced and over-subscribed. I also know I was lucky to have a strong support network around me – including a manager who cared about me so I did not lose my job and a husband who had committed many years before to always care for me so I didn’t lose anything more important than a job. I was never at risk of hurting myself or my baby. But I needed help to cope. I felt it wasn’t fair for me to let something that I could manage with help steal my quality of life. To steal happiness and joy from me and my children. Treatment is difficult – finding what works and what doesn’t isn’t easy. Actually, it’s fucking hard. It’s work. Hard work. But asking for help was the only way I could begin that journey and my doctor was able to quickly get me on the right track in time for my baby to be born.

There’s a lot of talk out there about post-natal depression – and there needs to be. But there isn’t much talk about antenatal and prenatal mental health. I was unwell during my first pregnancy but I put it down to being upset about how physically unwell I was and “mood swings”. I didn’t know it was possible to have antenatal depression or prenatal anxiety or any other pregnancy-related mental health issues. It was only the second time – when my illness became debilitating that I had a name for what I was going through. I wonder if I’d had a name for what I was going through the first time, I would have been more prepared the second time.

I wish I’d known, and I wish I’d sought help sooner. But mostly I’m glad that I could access help and that I did reach out. I’m grateful to my husband for helping me get the help I needed and my friends and manager for supporting me through the process.

It’s important to know that while it’s usual to feel blue occasionally or have ups and downs in your pregnancy – it’s not normal to feel overwhelmed *most* of the time, or to have more bad days than good.

When I look back, some of the thoughts and feelings I had showed I was unwell really clearly. I was obsessed with counting the movements of my baby because I thought he was dying inside me. I thought sleeping might hurt him so I used to try and stay awake all night. I was convinced he didn’t want me to be his mother. Clearly, they’re not the thoughts of a healthy person.

But other thoughts were subtle, and I want to share them with you because I want to suggest you talk to your midwife if you’re having any of these feelings while you’re pregnant.

I cried in the shower most days. I put this down to hormones. But actually, you shouldn’t cry that much while you’re pregnant. I put a lot down to hormones when what I was actually experiencing was depression and anxiety – feeling worried every day about finances, how we would manage, what kind of parent I would be with two children. It is normal to worry a little bit, it could be a sign of something bigger if you’re constantly worried. I felt emotionally numb a lot and sometimes didn’t even think about being pregnant. I felt it was hard to make a connection with my baby.

I had a lot of feelings about my upbringing. I think it’s normal to consider how you were raised when you’re about to start a family – but you should be able to process those fairly easily. If those feelings have a weight too heavy for you to carry, you should talk to someone.

Don’t let people tell you it’s “just hormones”. Talk to someone who actually knows what they’re talking about – a medical professional. It doesn’t matter if it’s your first pregnancy or your tenth – prenatal depression and anxiety can strike at any time.

If you feel like it’s impossible to find joy in being pregnant – talk to the person looking after you, your midwife, obstetrician, lead maternity carer – anyone with a medical background. They’ll be able to refer you to your GP or someone who can help work out whether what you’re feeling is standard pregnancy stuff, or something more serious. And if it’s more serious – that’s OK.

I felt like I’d already failed as a mother when I was put under maternal mental health. I thought I was a terrible mother who shouldn’t be allowed to have children. I thought horrible things about myself and actually considered that maybe I should just leave my husband to have both children – as I was so useless they wouldn’t even notice if I wasn’t there. I thought they’d be better off if I wasn’t there.

I struggle sometimes still with the guilt of being unwell at a time when I should have been happy, but I was once told by my doctor to imagine how I would view another mother who had sought help for mental health issues. He asked me to write down a message to her.

Dear mother,
You are brave.
You are strong.
It is a sign of your love for your children and your partner that you’re getting help so that you can be the best mother you can be.
It will be OK.

And it was.

**Content from Blog #17**

This past April, in [Location de-identified], I went out to dinner with some new girl friends. Our conversation that night became quite intimate and over the course of dinner I learned of one friend’s struggle with pregnancy depression. She shared, so eloquently, her struggle and journey to get support during her recent pregnancy. For the first time I found someone who I could open up to about my own experience with pregnancy depression. While I had spoken about it with close friends and family, this was the first time I could speak to someone who could completely relate. Her vivid accounts of her depression brought back memories of my own, and we found comfort in knowing that we weren’t alone with those struggles.

She drove me home that night and we discussed how pregnancy depression is so rarely spoken about – how it seems to be taboo – to be sad during what should be such a happy time of your life. I promised her that I would work up the courage to write about it. I’ve decided to share my personal experiences here to normalise something that, I’ve now learned, is not uncommon but is still rarely discussed.

I was 23 when I became pregnant with [Name de-identified]. I was young, carefree and madly in love with [Name de-identified]. I felt incredibly lucky to have found someone I wanted to spend the rest of my life with, to be starting a family and becoming a mother–a role that felt so natural to me having grown up with lots of younger siblings. I could not have been happier or more excited for what was to come.

And then, within weeks of becoming pregnant, I started having thoughts and feelings I had never experienced before. A wave of anxiety took hold of me, tightening its grip over my mind. For the first time in my life I could not control the thoughts that entered my head or shake the darkness out of my mind. I started thinking about death and how everyone I know will die some day. I obsessed about my grandparents and how they were getting older and how their lives would soon end. I couldn’t pass elderly people, even strangers, without feeling a tightening in my heart and tears welling up in my eyes. I thought about my own life and the new life I was creating. I thought about the generations of people who came before us and how there would be generations to come after us – the world so enormous and life so insignificant. I became so obsessed with these dark thoughts that I wasn’t able to find joy in the everyday life I was living. To add to this, I started feeling incredibly guilty – worried my thoughts would have a negative impact on my baby. I fretted that I was ruining the joy of pregnancy for both [Name de-identified] and myself.

I soon started worrying about [Name de-identified], obsessing about something terrible happening to him. I cried when he left for work every day with terrible, tragic scenarios playing out in my head. I tried to speak to Michael about it but I couldn’t even begin to explain it, and he couldn’t possibly understand. I wished I could play my thoughts on a projector for him, so that he could reassure me and tell me how silly they were.

A friend once told me that depression is a bit like staring at the sun. You know it’s not good for you. You know it will burn your eyes. But you can’t look away. The anxiety I experienced during pregnancy was exactly like this – I had thoughts running through my head that made me incredibly sad and worried, and yet I couldn’t turn away from them.

I remember towards the end of pregnancy, I was sitting with [Name de-identified] on a train up to [Location de-identified]. We were headed to [Location de-identified] for a fun work retreat with [Name de-identified] colleagues. I was 35 weeks pregnant, so close to meeting our baby. I sat on the train, staring out at the countryside quickly passing us by… and I started to panic. What was wrong with me? Why was I feeling this way? What if these feelings never went away? Or even worse, what if they got worse once the baby is born? I was aware of postpartum depression and worried that I might be affected by it. How could I be a decent mother if I was so sad and so emotionally unstable? Tears rolled down my cheeks as I stared out of that train, trying my hardest to pull myself together – trying to focus on something else, to think happy thoughts, to stop staring at the sun!

Five weeks later [Name de-identified] was born on a sunny June day, right on time. After a long labour he arrived quickly and beautifully, and his perfect tiny body was placed on my chest. Instantly, almost unbelievably, as if my mind had been simultaneously purged of its negative thoughts as I pushed my baby out, my mind returned to normal. Thoughts, both happy and sad, would come and go in the same normal, healthy way they did before I became pregnant. I felt joy like I’ve never felt in my life and, of course, immense relief to have control over my thoughts again. It is a testament to the power of hormones that a switch inside your mind can be triggered on with pregnancy and immediately switched off at pregnancy’s end.

When I was pregnant with [Name de-identified] I did not know that depression or anxiety could take place during pregnancy. I had heard about postpartum depression, but nobody had ever spoken about antenatal depression. I didn’t even think to look it up at the time. I just thought there was something wrong with me. And being a young, expectant mother in a new city, I didn’t have many friends to share my feelings with.

I experienced anxiety again when I became pregnant with [Name de-identified], but while I was affected with dark thoughts and feelings similar to those in my first pregnancy, I thankfully had the perspective and experience to assure myself that they were transient– that it would all go away when my baby was born. This basic understanding helped me to manage my depression during those nine months and again with [Name de-identified] birth, my mind and emotional state returned to normal as soon as he was born. Again, I was awed by the power of hormones and gained a deeper understanding and sympathy for those who suffer from postpartum depression or depression of any kind.

One week after speaking with my friend about her antenatal depression, I had a conversation with another friend who said her sister had also suffered severe depression during her pregnancy. It seemed the more I mentioned it, the more common I found it to be. I hope this post will start a conversation about a topic seldom discussed. Please share your thoughts and tell us if you’ve ever experienced depression before, during or after pregnancy and what helped you through this difficult time. As with so many aspects of the parenting journey, it is invaluable to learn from the experiences of other mothers and support each other along the way.

**Content from Blog #18**

I was one semester away from finishing graduate school when I decided that I wanted a baby NOW. I had always wanted one, but I suddenly became overwhelmed by an urgent need that I couldn't resist. I began to taper off the Celexa I had been on for years, and lowered my dose of Wellbutrin—all without the knowledge of my psychiatrist. I have had depression and anxiety my entire life but it had been under control for years, so I figured there would be no harm in me getting off one of my meds. I was feeling good and almost entirely off the Celexa when I took a pregnancy test and saw the two pinks lines. My husband and I were elated.

Two weeks later, I started to throw up. The nausea hit me like a tidal wave and it continued to swell, unrelenting. I threw up all day and night, rejecting nearly everything I ingested. The sight of a glass of water made me gag. For two weeks straight I consumed nothing but Gatorade and Cheerios. I was also suffering from severe insomnia and it became clear that there was no way I would be able to finish my final semester of school, which would have involved full-time student teaching.

With no job and no school, I stayed home all day by myself, puking and crying and wishing I were dead. In my ninth week of pregnancy, the vomiting became so relentless that I went to the hospital for an IV. It was the worst night of my life. I was buried deeper in despair than I thought humanly possible and realized the only way for me to escape would be to either terminate my pregnancy or terminate my life. This thought gave me the only feeling of hope or peace I'd had in weeks. I eyed a bottle of klonopin in my medicine cabinet with excitement. It could be a way out for me.

When I left the hospital, I begged my husband to let me have an abortion. He looked at me with horror and told me it wasn't an option. I had never felt so enraged. Why did I have to go through this, alone? It was my body, MY suffering. That night after he went to bed, I looked up the information for my local Planned Parenthood chapter and decided I would have the abortion without my husband's knowledge. I didn’t think about how I would explain to him how I was no longer pregnant. All I thought of was escape and the idea of it was glorious. I got in bed and lay awake all night, as had become my new pattern, fantasizing about my abortion—my yearning to end the pregnancy I had wanted my entire life. When I got out of bed to vomit, as was my other pattern, I had my first hallucination. It was the voice of a child saying "Hi!" The sound of this voice was so loud and clear it made me jump.

The next morning, after having my second hallucination (this time it was the terrifying vision of my husband holding a baby on his hip), I became hysterical and told my husband I was going to kill myself. He called my mother and sister and they took me straight to a psychiatric hospital. By that point, I was in such hell that I felt relieved to be taken somewhere—anywhere—other than where I had been over the previous few weeks, suffering terribly.

For the next seven days, I attended group therapy sessions, stabilized my medication, meditated, read, slept, colored with crayons, played Monopoly, and watched a 60-year-old schizophrenic woman strip naked and run through the hallways screaming. I met people who were terribly ill, and others who had reached low points that had become dangerous. We cried and laughed together at our common madness. There was humor in it, and great sadness. My husband and sister came to visit me every day, and other family members came, too. As I started to feel better physically and emotionally, I focused on taking things one day at a time and being grateful for everything I had: a supportive family and friends, a husband who loved and accepted me no matter what, and the baby growing inside of me.

After leaving the hospital, I started to see an amazing therapist and continued my path to healing, albeit slowly. Things got better, but I still struggled with severe nausea and my depression, though greatly improved, lingered a bit. I felt sad about giving up graduate school and felt lonely staying home by myself during the day. As I entered my third trimester, the severity of my nausea diminished and I took on some freelance work to keep me busy. I became more and more attached to the little baby wiggling inside of me. I dreamed of what she would look like and what her skin would feel like, and I put ultrasound photos on the refrigerator.

As my due date neared, my psychiatrist and I, along with my OB-GYN, decided I would be best off not breast-feeding so I could go on additional medication and not have to deal the hormonal fluctuations and frustrations that come with nursing. I felt guilty for not being able to breastfeed, but was reminded by my amazing doctors that my own health and wellbeing was paramount if I wanted to be the best mother possible. And I did. "Healthy mom equals healthy baby," they said, and I understood that that was true.

After 9 long months—11 days past my due date—I finally gave birth to a beautiful baby girl. She is the great love of my life and after I held her for the first time, the purpose of all my suffering made sense. It was a shifting storm that gave way to a beautiful, clear sky. When I think of how badly I wanted to end my pregnancy I still feel guilty, but then I remind myself that I was sick and beyond my capacity to cope. I needed help to get to a place of peace and healing—there is no way I could have done it on my own. I have nothing but gratitude for the doctors who took care of me and my loved ones who supported me every step of the way. And most of all, the beautiful baby girl who saved my life.
